# The geranyl acetophenone tHGA attenuates human bronchial smooth muscle proliferation via inhibition of AKT phosphorylation

**DOI:** 10.1038/s41598-018-34847-0

**Published:** 2018-11-09

**Authors:** Hui Min Yap, Yu Zhao Lee, Hanis Hazeera Harith, Chau Ling Tham, Manraj Singh Cheema, Khozirah Shaari, Daud Ahmad Israf

**Affiliations:** 1 Department of Biomedical Science, Faculty of Medicine and Health Sciences, Universiti Putra Malaysia, 43400 Serdang, Selangor Malaysia; 2 Institute of Bioscience, Universiti Putra Malaysia, 43400 Serdang, Selangor Malaysia

## Abstract

Increased airway smooth muscle (ASM) mass is a prominent hallmark of airway remodeling in asthma. Inhaled corticosteroids and long-acting beta_2_-agonists remain the mainstay of asthma therapy, however are not curative and ineffective in attenuating airway remodeling. The geranyl acetophenone 2,4,6-trihydroxy-3-geranyl acetophenone (tHGA), an in-house synthetic non-steroidal compound, attenuates airway hyperresponsiveness and remodeling in murine models of asthma. The effect of tHGA upon human ASM proliferation, migration and survival in response to growth factors was assessed and its molecular target was determined. Following serum starvation and induction with growth factors, proliferation and migration of human bronchial smooth muscle cells (hBSMCs) treated with tHGA were significantly inhibited without any significant effects upon cell survival. tHGA caused arrest of hBSMC proliferation at the G_1_ phase of the cell cycle with downregulation of cell cycle proteins, cyclin D1 and diminished degradation of cyclin-dependent kinase inhibitor (CKI), p27^Kip1^. The inhibitory effect of tHGA was demonstrated to be related to its direct inhibition of AKT phosphorylation, as well as inhibition of JNK and STAT3 signal transduction. Our findings highlight the anti-remodeling potential of this drug lead in chronic airway disease.

## Introduction

Airway remodeling, a collective term describing the structural changes in the asthmatic airway, occurs in conjunction with, or as a result of, chronic airway inflammation^1,2^. The asthmatic airway undergoes remodeling as a healing process which involves increased airway smooth muscle (ASM) mass, sup-epithelial fibrosis, epithelium mesenchymal transition (EMT), goblet cell and myofibroblast hyperplasia^2–4^. As a consequence of these structural changes, thickening of the airway wall causes lumen narrowing that ultimately leads to airway obstruction^4^.

Current asthma treatment regimens employ a combination of inhaled corticosteroids (ICS) and beta_2_-agonists that provide minimal beneficial effects upon airway remodeling^5,6^. It has been suggested that airway remodeling may not be reversed by steroid treatment but rather prevented^7^. Hence there seems to be alternative molecular targets that may be directly responsible for airway remodeling which are independent of proinflammatory processes. Furthermore, repeated allergen challenge in murine models have been shown to result in persistent airway remodeling following resolution of airway inflammation and hyperresponsiveness (AHR)^8,9^. Hence, treatments that target single or multiple components of pathways that induce airway remodeling may be useful in the management of asthma.

Our previous studies demonstrated that 2,4,6-trihydroxy-3-geranyl acetophenone (tHGA) is effective in attenuating AHR in response to methacholine challenge as well as reducing inflammatory cell infiltration in both acute and chronic murine models of asthma^10,11^. Furthermore, tHGA-treated mice were found to have reduced expression of α-SMA and thinner layers of smooth muscle surrounding the airways in comparison to untreated mice^11^. Thickening of the airway wall, primarily due to increased ASM mass, reduces the diameter of the airway as it contracts and causes significant airflow limitation and AHR^12^. Another study of ours recently demonstrated that tHGA attenuated eosinophil-induced epithelial-mesenchymal transition (EMT) of bronchial epithelial cells in a concentration-dependent fashion through its suppression of transforming-growth factor-β (TGF-β) synthesis via both PI3K and JNK pathways^13^. Hence, we are interested to explore further the pharmacological effects of tHGA in modulating various elements of tissue remodeling.

ASM mass is increased through hyperplasia and hypertrophy^12^. ASM hyperplasia can be defined as an increased number of ASM cells in the asthmatic airway. This increase in cell number is either due to increased cellular proliferation, reduced apoptosis or/and increased cellular migration towards the airway lumen in response to proinflammatory mediators release^14,15^. Proinflammatory mediators such as growth factors and cytokines activate several signal transduction pathways through binding to tyrosine kinase receptor (RTK) and G protein-coupled receptors (GPCRs) that culminate in proliferation and migration of ASM^16–19^. In this communication, we describe the inhibitory effect of tHGA upon growth factor-induced ASM cell proliferation and migration in an established cellular model. This effect was found to be related to the inhibition of AKT phosphorylation, a downstream signaling molecule of the PI3K pathway that plays a regulatory role in smooth muscle cell proliferation, migration and apoptosis^20,21^.

## Results

### tHGA inhibits growth factor-induced human bronchial smooth muscle cell (hBSMC) proliferation and migration

**Figure 1:**
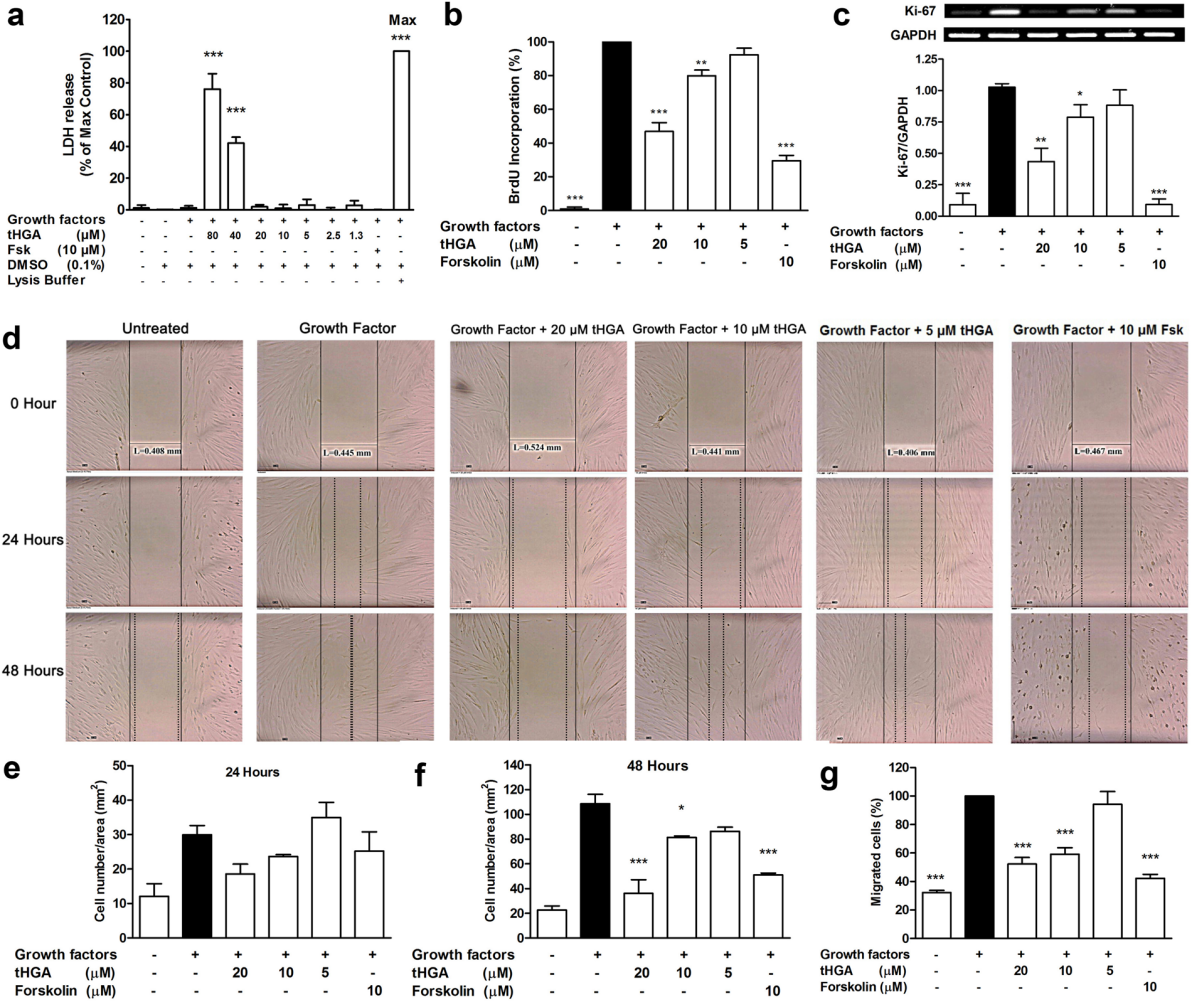
tHGA inhibits growth factor-induced hBSMCs proliferation and migration. hBMSCs were induced with growth factors and co-treated with tHGA or forskolin for 48 hours. (**a**) Non-cytotoxic concentration of tHGA on growth factor-induced hBSMCs was assessed through the release of LDH. Proliferation of growth factor-induced hBSMCs upon tHGA or forskolin co-treatment for 48 hours was measured through (**b**) BrdU proliferation assay and (**c**) Ki-67 mRNA expression study. Cell proliferation is presented in percentage (%). Expression of Ki-67 mRNA was normalized to internal control, GAPDH. Representative cropped gel images for Ki-67 are presented (full-length gel is presented in Supplemental Fig. S3). (**d**) Representative images of scratch assay at 0, 24 and 48 hours after co-treatment with growth factors and tHGA or forskolin. The number of cells migrated was normalized to the scratch area (mm^2^) after 24 (**e**) and 48 (**f**) hours co-treatment with growth factors and tHGA or forskolin. (**g**) Percentage of cells migrated to the lower chamber in transwell migration assay after 6 hours of co-treatment between growth factors and tHGA or forskolin. Results are presented as means ± SEM of 3 independent experiments. *P < 0.05, **P < 0.01 and ***P < 0.001, significantly different from growth factor-induced hBSMCs. Fsk: Forskolin; DMSO: Dimethyl sulfoxide.

To determine the maximum non-cytotoxic concentration of tHGA for further experiments, lactate dehydrogenase (LDH) release from growth factor-induced hBSMCs following tHGA treatment was measured. tHGA concentrations of 20 µM and below were not cytotoxic (Fig. 1a), and therefore used for subsequent experiments. Forskolin (10 µM) and the vehicle 0.1% dimethyl sulfoxide (DMSO) did not induce any significant LDH release.

Growth factor-enriched media induced a significant increase (*P* value < 0.001) in the percentage of bromodeoxyuridine (BrdU)-positive hBSMCs after 48 hours compared to non-induced hBSMCs. Co-treatment with tHGA showed a significant decrease in BrdU-positive cells at 20 µM (47 ± 5.2%) and 10 µM (80 ± 3.4%) compared to growth factor-induced hBSMC (100%) (Fig. 1b). tHGA was shown to suppress growth factor-induced hBSMC proliferation in a concentration-dependent manner. The anti-proliferative effect of tHGA upon growth factor-induced hBSMCs was further validated by assessing the changes in the gene expression of the proliferation marker Ki-67. A similar trend was observed in which induced hBSMCs showed a 10-fold increase in Ki-67 mRNA expression which was significantly inhibited with 20 µM and 10 µM with tHGA (Fig. 1c). Forskolin (10 µM), which served as the positive assay control, significantly reduced growth factor-induced hBSMC proliferation which correlated with reduced Ki-67 mRNA expression.

Migration of hBSMCs in both scratch and transwell migration assays are shown in Fig. 1d–g. In the scratch assay, treatment with growth factors for 24 hours did not induce significant migration of hBSMCs (Fig. 1e). However, significant increase in cell migration was observed after 48 hours incubation time (Fig. 1f). tHGA significantly inhibited the migration of induced hBSMCs by approximately 67 ± 11% at 20 µM in the scratch assay (Fig. 1f). Transwell assay demonstrated that growth factor significantly increased the percentage of hBSMCs migration to the lower chamber after 6 hours of incubation time. tHGA, at 20 µM, inhibited approximately 48 ± 5% of hBSMCs migration to the lower chamber (Fig. 1g).

### tHGA causes hBSMC arrest at the G_1_ cell cycle phase without inducing apoptosis

**Figure 2:**
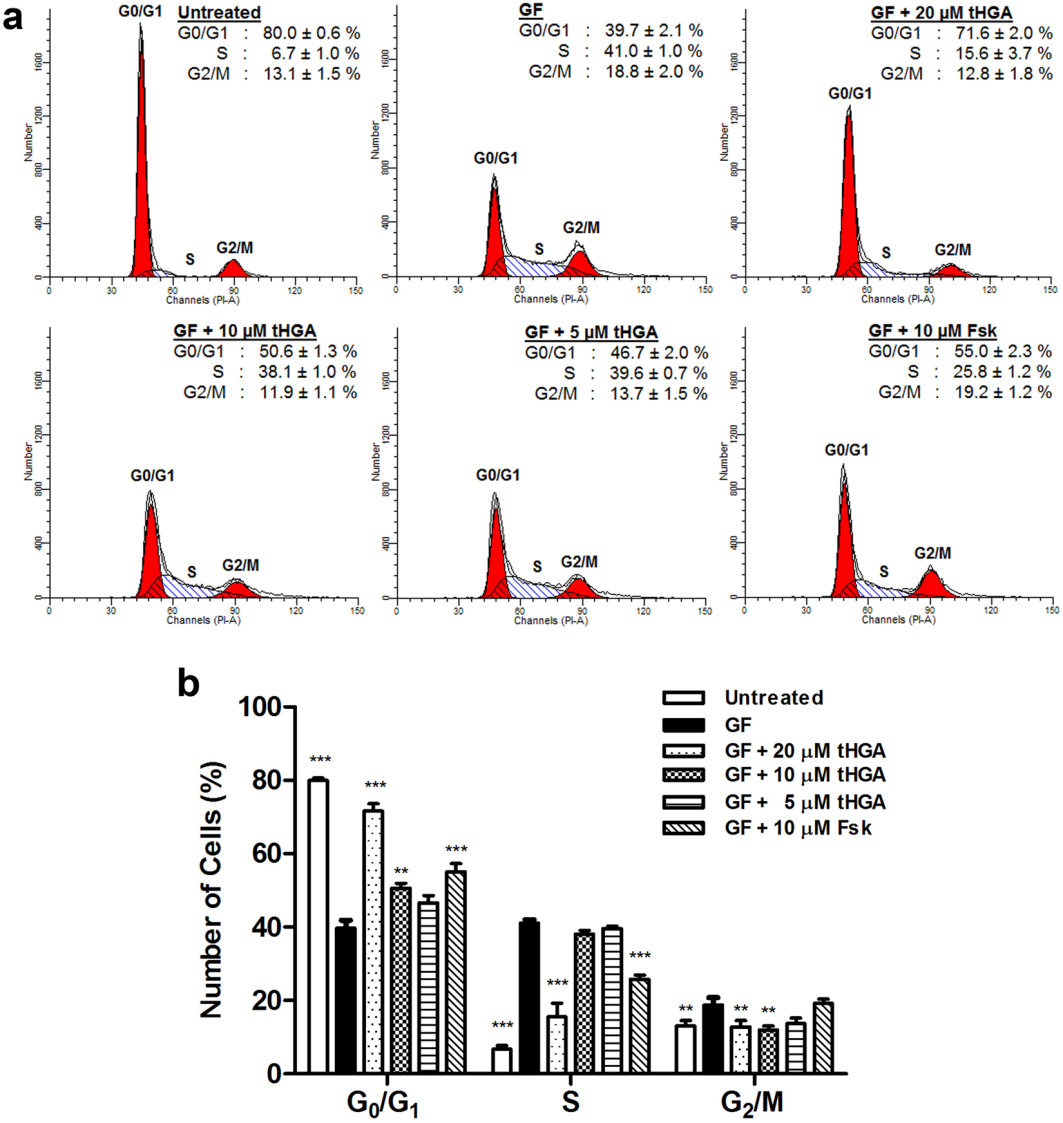
tHGA arrests growth factor-induced hBSMCs proliferation at G_0_/G_1_ phase. hBSMCs were induced with growth factors and co-treated with tHGA or forskolin for 48 hours. (**a**) Representative histograms of cell cycle analysis on growth factor-induced hBSMCs upon tHGA or forskolin treatment. (**b**) Percentages of hBSMCs in different cell cycle stages following indicated treatments.

To further understand the mechanism of tHGA in inhibiting the proliferation of hBSMCs, its effect upon cell cycle regulation was assessed. Cell cycle analysis demonstrated that growth factors induced a significant increase in the percentage of hBSMCs entering the S phase as compared to non-induced hBSMCs (non-induced: 7 ± 1.0% *vs* induced: 41 ± 1.0%) (Fig. 2a,b). A significant reduction in the percentage of hBSMCs entering the S phase was observed following co-treatment with growth factors and 20 µM tHGA. The percentage of induced hBSMCs accumulated at the G_1_ phase following tHGA treatment (20 µM and 10 µM) was significantly higher than that observed in untreated induced hBSMCs. The assay control forskolin also caused a significant increase in the percentage of cells in the G_1_ phase and reduced the percentage of cells in the S phase in comparison to induced cells.

**Figure 3:**
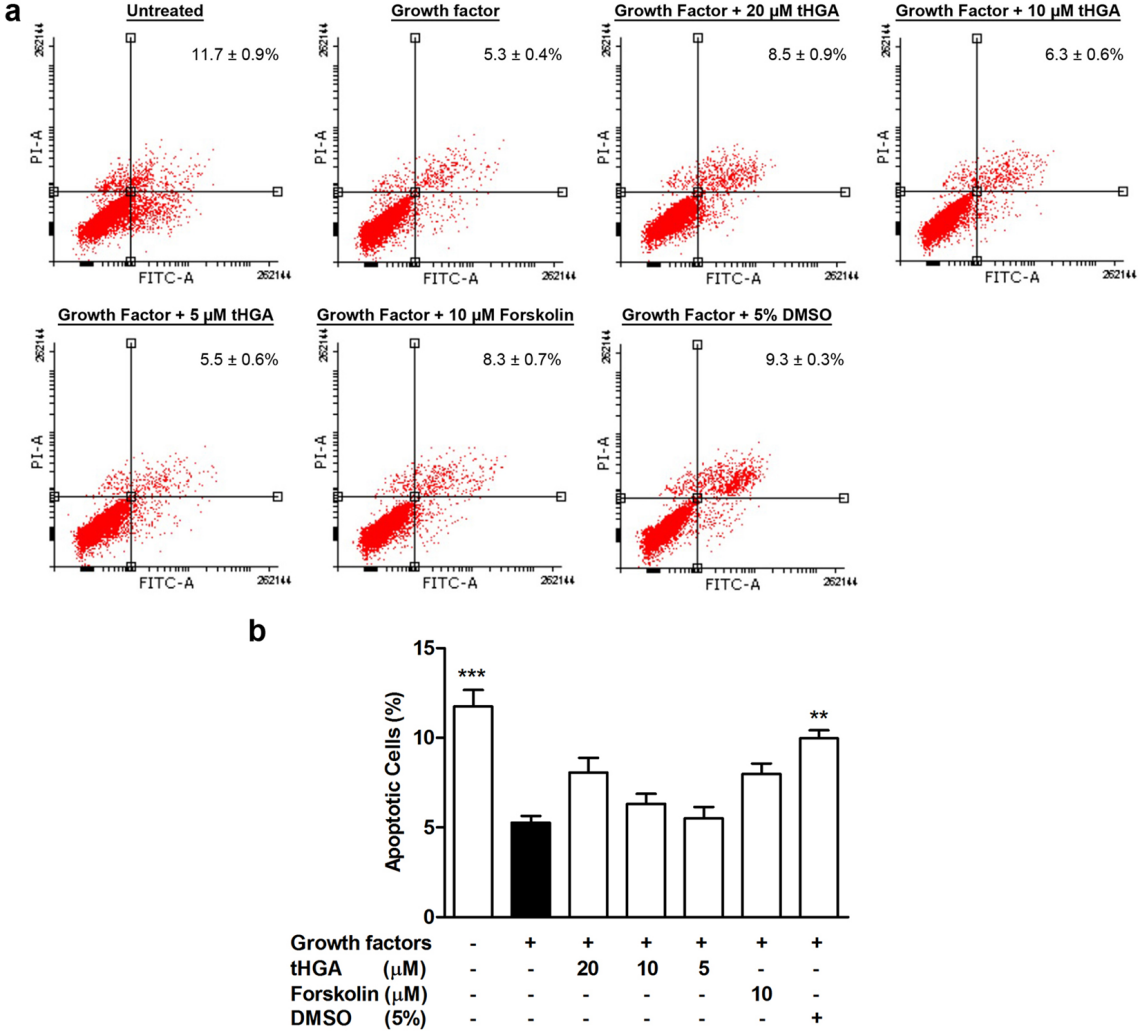
tHGA attenuates growth factor-induced hBSMC proliferation without inducing apoptosis. (**a**) Representative dot plots of Annexin V and PI flow cytometry apoptosis analysis on growth factor-induced hBSMCs upon tHGA or forskolin treatment. (**b**) Percentage of apoptotic hBSMCs upon tHGA or forskolin treatment. Results are presented as mean ± SEM of 3 independent experiments. *P < 0.05, **P < 0.01 and ***P < 0.001, significantly different from growth factor-induced hBSMCs. GF: Growth factor; Fsk: Forskolin.

Since ASM hyperplasia could be due to increased cell proliferation or reduced apoptosis, the effect of tHGA upon hBSMC apoptosis following growth factor induction was also examined. Non-induced hBSMCs demonstrated a higher percentage (11.7 ± 0.9%) of apoptotic cells as compared to induced hBSMCs (5.3 ± 0.6%). Conversely, 20 µM tHGA caused a slight, yet insignificant increase (8.5 ± 1.6%) in the percentage of apoptotic cells (Fig. 3a,b). Similarly, forskolin caused a slight increase (insignificant) of apoptotic cells in growth factor-induced hBSMCs from 5.3 ± 0.6% to 8.0 ± 1.0%. The assay control DMSO (5%) significantly increased the percentage of apoptotic cells in growth factor-induced hBSMCs.

### Effects of tHGA upon cell cycle regulators and signaling molecules following growth factor induction

#### tHGA attenuates cyclin D1 and p27^Kip1^ following growth factor induction

**Figure 4:**
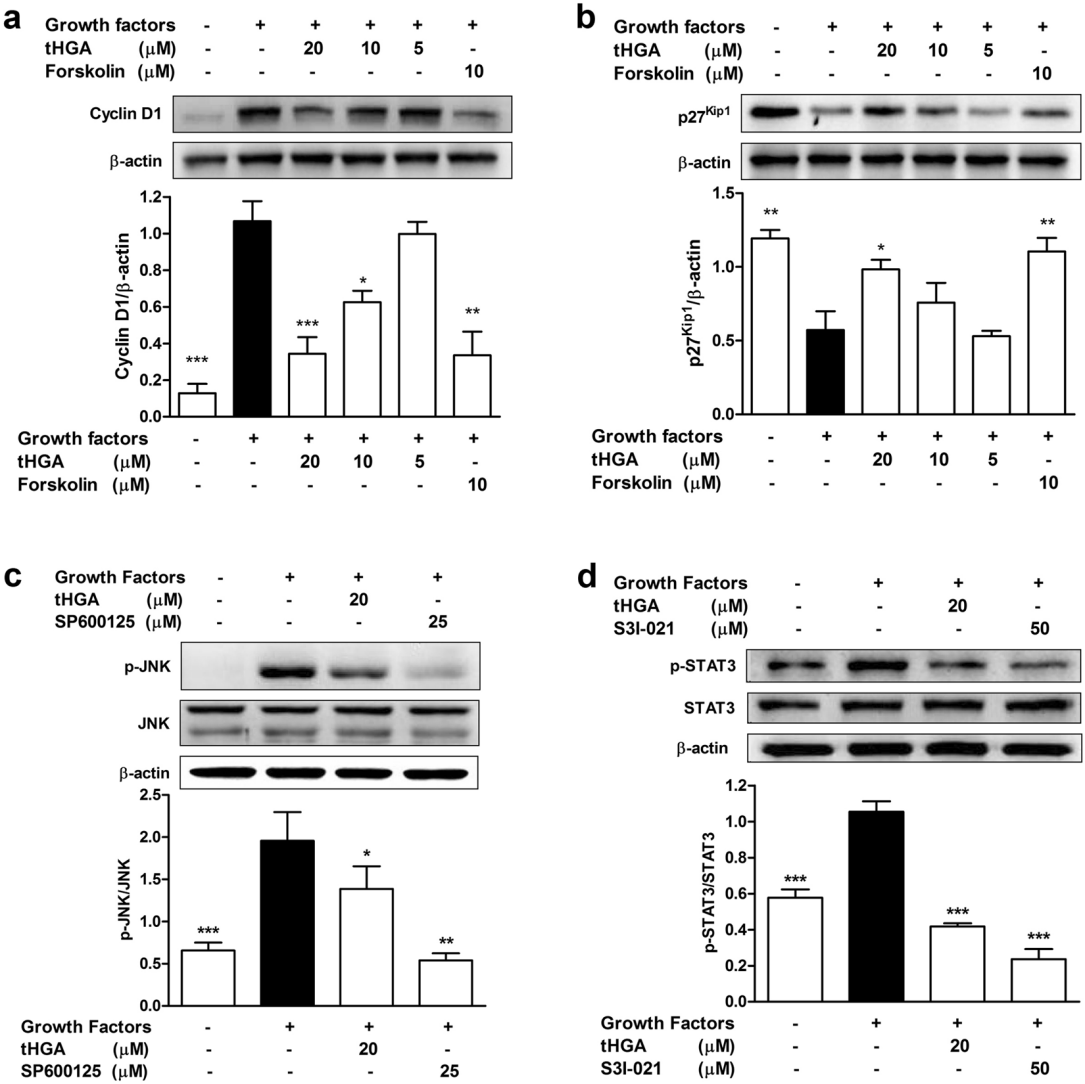
Effects of tHGA on the expression of cell cycle proteins and phosphorylation of proliferation signaling molecules in growth factor-induced hBSMCs. hBSMCs were co-treated with growth factors and tHGA or forskolin for 20 hours, followed by analysis of (**a**) cyclin D1 and (**b**) p27^Kip1^ protein expression through immunoblotting. Protein expression of cyclin D1 and p27^Kip1^ were normalized to the internal loading control β-actin. Phosphorylation of (**c**) JNK and (**d**) STAT3 in growth factor-induced hBSMCs upon tHGA treatment were assessed through immunoblotting. hBSMCs were serum-starved overnight before co-treatment with growth factors and tHGA or respective inhibitors (SP600125 or S3I-201) for 1 hour. Proteins were immunoblotted with respective antibodies and the protein bands were quantified by densitometry. Results of (**c**) phospho-JNK and (**d**) phospho-STAT3 were presented after normalization with total JNK and STAT3 respectively. Representative cropped blots are presented (full-length blots are presented in Supplementary Fig. S4). Results are presented as mean ± SEM of 3 independent experiments. *P < 0.05, **P < 0.01 and ***P < 0.001, significantly different from growth factor induced-hBSMCs.

Cyclin D1 and p27^Kip1^ are key regulators of G_1_/S progression in hBSMCs^22^. As cells progress through the G_1_ phase, cyclin D1 is rapidly expressed in response to growth factors. Conversely, p27^Kip1^, a cyclin kinase inhibitor, will be degraded during the G_1_ phase^23^. Therefore, expression of cyclin D1 and p27^Kip1^ were examined to determine whether tHGA had any effect upon these key regulatory molecules. tHGA significantly inhibited growth factor-induced cyclin D1 expression in hBSMCs at 20 and 10 µM (Fig. 4a). Conversely, p27^Kip1^ expression was reduced in hBSMCs in response to growth factor induction. However, this was reversed by 20 uM tHGA treatment, an effect that was comparable to forskolin (Fig. 4b).

#### Effects of tHGA on MAPK signaling molecules in growth factor-induced hBSMCs

ERK1/2, JNK and p38 are the three main signaling molecules in MAPK pathway^24^. Growth factors significantly induced phosphorylation of JNK (Fig. 4c), ERK1/2 (see Supplementary Fig. S1a) and p38 (see Supplementary Fig. S1b) in hBSMCs following 1 hour. tHGA caused a significant inhibition of JNK phosphorylation as shown in Fig. 4c. However, tHGA failed to inhibit the phosphorylation of ERK1/2 and p38 (see Supplementary Fig. S1). All MAPK inhibitor controls caused significant inhibition in MAPK phosphorylation.

#### Effects of tHGA on JAK2/STAT3 signaling molecules in growth factor-induced hBSMCs

Cyclin D1 and p27^Kip2^ in ASM are mediated through JAK2/STAT3, MAPKs and PI3K/AKT signaling pathways, which are known to regulate several important cellular activities, including cell proliferation, migration and survival^25–27^. Since tHGA demonstrated inhibition of cyclin D1 as well as p27^Kip2^ degradation, the effect of tHGA upon these signaling pathways was further examined. Induction with growth factors for 1 hour caused a significant increase in JAK2 phosphorylation. Growth factor-induced JAK2 phosphorylation was significantly inhibited in the presence of JAK2 inhibitor (AG490). However, tHGA failed to exert any significant effect (see Supplementary Fig. S1) although it did seem to cause complete inhibition of STAT3 (downstream molecule of JAK2) phosphorylation similar to the STAT3 inhibitor S3I-021 (Fig. 4d).

#### Effects of tHGA on PI3K signaling molecules in growth factor-induced hBSMCs

**Figure 5:**
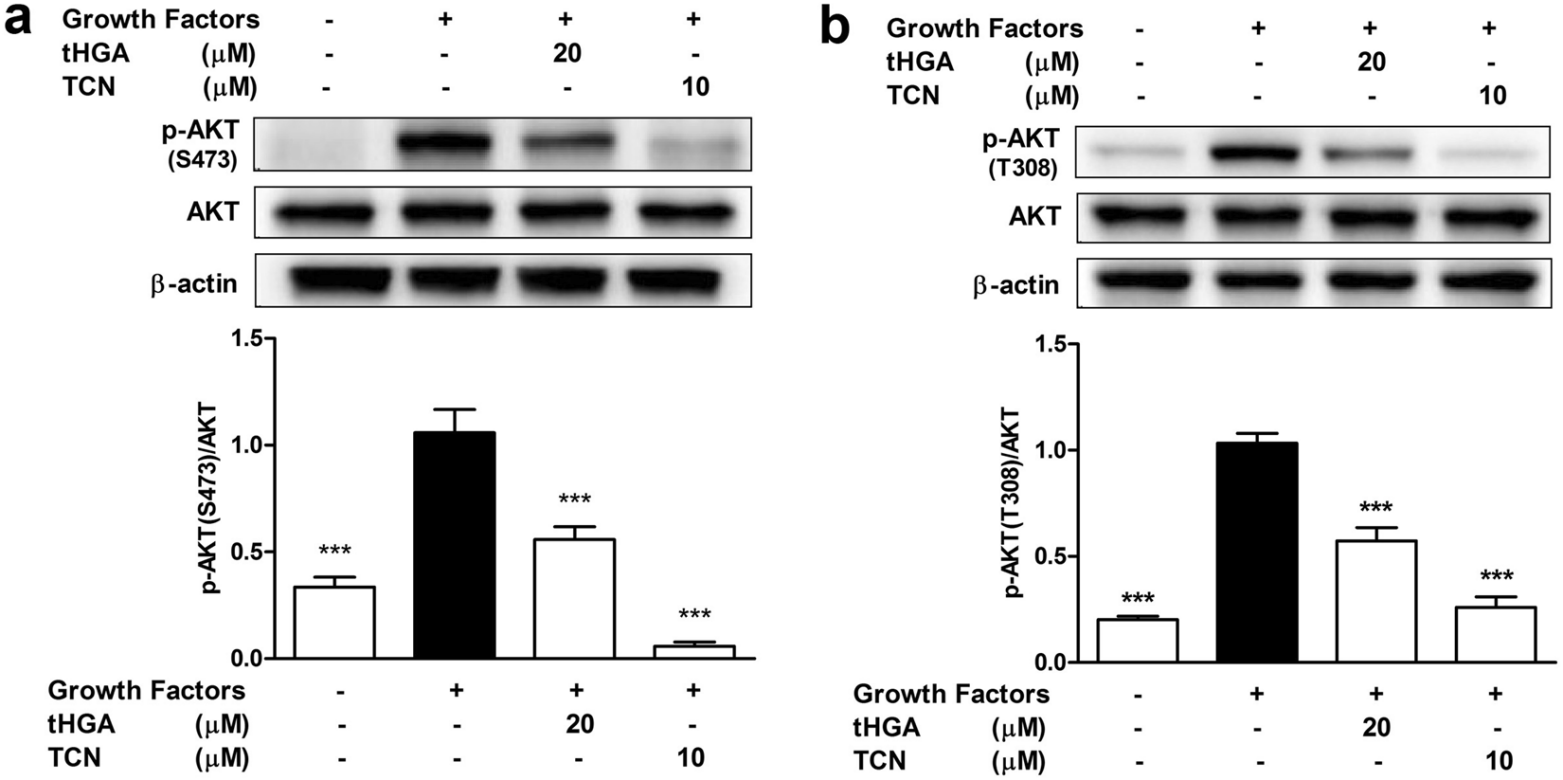
Effects of tHGA on the phosphorylation of AKT in growth factor-induced hBSMCs. hBSMCs were serum-starved overnight before co-treatment with growth factors and tHGA or triciribine (TCN) for 1 hour. Phosphorylation of (**a**) AKT (Ser473) and (**b**) AKT (Thr308) in growth factor-induced hBSMCs upon tHGA treatment were assessed through immunoblotting. Proteins were immunoblotted with respective antibodies and the protein bands were quantified by densitometry. Results of (**a**) phospho-AKT (Ser473) and (**b**) phospho-AKT (Thr308) were presented after normalization with total AKT. Representative cropped blots are presented (full-length blots are presented in Supplementary Fig. S4). Results are presented as mean ± SEM of 3 independent experiments. *P < 0.05, **P < 0.01 and ***P < 0.001, significantly different from growth factor induced-hBSMCs. TCN: Triciribine.

Growth factors significantly increased the phosphorylation of PI3K after 1 hour of induction (see Supplementary Fig. S2a). However, tHGA did not reverse growth factor-induced phosphorylation of PI3K. Further investigations were carried out to examine the downstream molecules of PI3K, which include PDK1 and AKT. Growth factors induced a significant increase in the phosphorylation of PDK1 after 1 hour of induction without any significant effect of tHGA (see Supplementary Fig. S2b). However, tHGA demonstrated a significant reduction in growth factor-induced AKT phosphorylation at both Ser473 (Fig. 5a) and Thr308 (Fig. 5b). A similar trend was observed with the AKT inhibitor triciribine (Fig. 5a,b).

Since tHGA affected the phosphorylation of AKT without affecting the phosphorylation of upstream molecules, it can be postulated that AKT could be the potential molecular target of tHGA. In order to evaluate AKT as the potential molecular target of tHGA, key regulatory components that are involved in the phosphorylation of AKT were studied. In response to growth factor, mTORC2 will phosphorylate AKT at the Ser473 phosphorylation site, while PDK1 will phosphorylate AKT at the Thr308 phosphorylation site^28,29^. Therefore, further analysis focused upon the possibility of tHGA acting upon PDK1 and mTORC2 kinase activity. Kinase activity of PDK1 or mTORC2 following growth factor stimulation or tHGA treatment was determined through its capacity to induce *in vitro* phosphorylation of AKT (inactive), which served as the substrate for PDK1 and mTORC2. mTORC2 was immunoprecipitated from the protein lysate by using an anti-Rictor antibody; Rictor is an essential component of mTORC2^28^. The integrity of the mTORC2 complex was preserved following tHGA treatment as shown by the presence of mTOR, which was detected with an anti-mTOR antibody (see Supplementary Fig. S2c). Growth factor stimulation revealed a significant increase in kinase activity of mTORC2 as demonstrated by a significant increase of *in vitro* AKT (Ser473) phosphorylation (see Supplementary Fig. S2c). tHGA failed to inhibit mTORC2 kinase activity as demonstrated by the *in vitro* phosphorylation of AKT (Ser473). LY294002, which is PI3K inhibitor, significantly reduced kinase activity of mTORC2.

Growth factors significantly increased the kinase activity of PDK1 as demonstrated by an increase of *in vitro* AKT(Thr308) phosphorylation (see Supplementary Fig. S2d). Similarly, tHGA failed to attenuate the effect of growth factors upon PDK1 kinase activity. Meanwhile, BX-795 showed significant inhibition on the kinase activity of PDK1 as demonstrated by a significant decrease of the *in vitro* phosphorylation of AKT (Thr308).

#### Effects of tHGA on myr-AKT transfected hBSMCs

**Figure 6:**
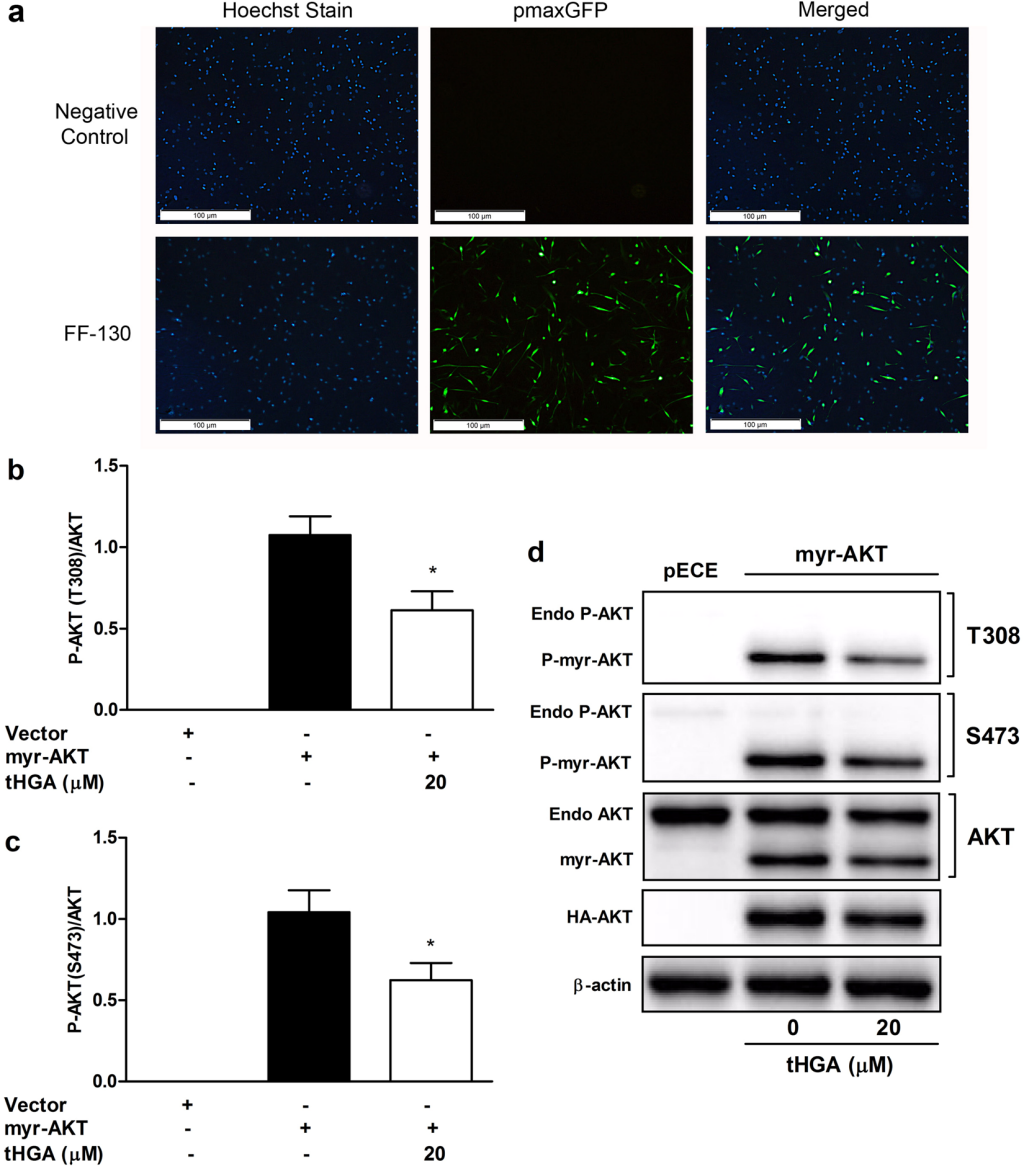
tHGA attenuates constitutively active AKT in hBSMCs. (**a**) hBSMCs were transfected with pmaxGFP with negative control (no program) or Nucleofection program FF-130. Transfected hBSMCs were seeded in complete media for 48 hours and then stained with Hoechst stain. Representative images of hBSMCs post transfection were depicted. (**b**,**c**) hBMSCs were transfected with pECE empty vector or myr-Akt and seeded in complete media for 24 hours prior to treatment. Transfected hBSMCs were then serum starved for 24 hours before treated with/without 20 µM tHGA for 1 hour. Proteins were immunoblotted with respective antibodies and the protein bands were quantified by densitometry. Results of (**b**) phospho-AKT (Thr308) and (**c**) phospho-AKT (Ser473) were presented after normalization with total AKT. (**d**) Representative cropped blots for results (**b**,**c**) are depicted (full-length blots are presented in Supplementary Fig. S4). Results are presented as mean ± SEM of 3 independent experiments. *P < 0.05, **P < 0.01 and ***P < 0.001, significantly different from growth factor induced-hBSMCs. Endo: Endogenous.

Since tHGA demonstrated an inhibitory effect upon AKT phosphorylation without any effect upon upstream molecules, it is likely that AKT may act as a potential target of tHGA. Therefore, we attempted to validate the role of AKT as a target for tHGA by using a constitutively active AKT construct (myr-AKT). Transfection conditions were optimized with a positive control pmaxGFP. hBSMCs transfected with program FF-130 demonstrated a 72% transfection efficiency and 86% cell viability, hence program FF-130 was used for subsequent transfections (Fig. 6a). HA-tagged myr-AKT in hBSMCs was detected with HA-tag antibody at approximately 50 kDa through immunoblotting. tHGA (20 µM) demonstrated a significant inhibition of the phosphorylation of myr-AKT at Thr308 (Fig. 6b) as well as Ser473 (Fig. 6c). This finding suggests that tHGA interferes with the myristoylation signal of myr-AKT by binding to AKT, thus inhibiting the phosphorylation of AKT and its activation.

## Discussion

Airway remodeling has been recognized as a major factor contributing to AHR and reduced lung function^30^. Increased ASM mass, a hallmark of airway remodeling, causes airway narrowing and airflow obstruction^31^. The effect of the most widely used asthma therapies, corticosteroids and β_2_-agonists, in reducing the increased ASM mass remains controversial^32^. Therefore, treatment that can prevent or reverse airway remodeling is much sought after in the management of asthma. Our previous study revealed that tHGA exhibited significant inhibitory effects upon AHR as well as airway remodeling in a chronic murine model of asthma. In particular, treatment of tHGA was shown to inhibit accumulation of the ASM layer, which is demonstrated by reduced expression of α-SMA in chronic ovalbumin (OVA)-induced asthmatic mice^11^. In this study, an attempt was made to determine whether tHGA had any effect upon the proliferation/migration/apoptosis of bronchial smooth muscle cells and to dissect the mechanism of action.

tHGA was shown to attenuate ASM proliferation induced by growth factors as indicated in the BrdU proliferation assay. This was further supported by the significant decrease in the expression of proliferation marker, Ki-67 following tHGA treatment, providing a strong indication of the anti-proliferative role of tHGA in growth factor-induced ASM proliferation. To further understand the underlying mechanism of tHGA action, three major pathways relevant to proliferation, namely MAPKs, JAK2/STAT3 and PI3K were studied. It was found that tHGA inhibited the phosphorylation of several key pathway regulators, namely JNK, STAT3 and AKT.

The JAK2/STAT3 pathway has been described recently as another important pathway involved in the development of allergic asthma^33^. However, tHGA only inhibited the phosphorylation of STAT3 while not affecting JAK2 phosphorylation. STAT3 inhibition was reported to attenuate house dust mite (HDM)-induced airway inflammation and remodeling in a murine model of asthma^34^. The inhibition of STAT3 phosphorylation by tHGA highlights its anti-proliferative potential since knockdown of STAT3 expression in ASM has been reported to significantly reduce ASM proliferation^35^. Several reports have demonstrated that activation of the MAPKs and PI3K pathways will induce the activation of transcription factors, including STAT3, that are required for cyclin D1 expression^36–38^. This study demonstrated reduced cyclin D1 expression following tHGA treatment. This study demonstrates that tHGA inhibits STAT3 phosphorylation, but did not inhibit the phosphorylation of JAK2, a STAT3 upstream activator. Therefore, further investigations are required to determine whether tHGA’s inhibitory effect on ASM proliferation may also involve its inhibitory effect on other upstream activators of STAT3 including PI3K or MAPKs^39^.

Amongst the MAPKs, the ERK pathway is suggested to dominate the regulation of ASM proliferation^40^. In the case of tHGA, it was demonstrated that despite no effect whatsoever upon ERK expression, the treatment was still able to induce an anti-proliferative effect. A recent study demonstrated the role of JNK in ASM proliferation, revealing that this pathway regulates human ASM proliferation partly through its regulation of cyclin D1 and cell cycle progression^25^. This suggests that JNK inhibition by tHGA could be one of the factors for the reduced level of cyclin D1 in our current findings. Together with MAPKs, PI3K is another main signaling pathway activated by mitogenic stimuli in ASM. tHGA showed significant inhibition upon AKT phosphorylation, a downstream signaling molecule of the PI3K pathway that plays a critical role as a regulator in cell proliferation, migration and apoptosis^41^. Upon mitogenic stimulation, PI3K phosphorylates phosphatidylinositol membrane lipids, thus producing the second messenger phosphatidylinositol-3,4,5-triphosphate (PIP_3_)^42^. AKT will be recruited to the plasma membrane by PIP_3_ where its conformation is altered to allow subsequent phosphorylation by PDK1 and cell cycle progression through regulation of cyclin D1 and p27^Kip1^ expression^43,44^.

Based on the findings obtained, the identification of the molecular targets of tHGA was narrowed down to either AKT or JNK. This is not surprising since there is crosstalk between both PI3K and MAPK pathways^45^. A model employing dominant-negative AKT transfected human bronchial epithelial cells demonstrated AKT acts as an upstream regulator of JNK in arsenite-mediated proliferation via PI3K/AKT/JNK/c-Jun/cyclin D1 signaling^46^. Another study using PTEN null cells demonstrated JNK and AKT to be independently activated by PI3K, indicating that PI3K instead of AKT acted as the upstream effector of JNK^47^. These findings point to a possible inhibitory effect of tHGA upon PI3K or AKT activation leading to the inhibition of JNK phosphorylation, thus making JNK more of a downstream effector instead of the molecular target of tHGA.

Since the major inhibitory effect of tHGA was upon AKT phosphorylation, effects upon upstream molecules of AKT, including PI3K, PDK1 and mTORC2, were investigated. The specificity of AKT as a target is suggested since no effects upon phosphorylation of PI3K and PDK1 as well as the kinase activities of PDK1 and mTORC2 was shown. Inhibition of AKT is attained through various ways, namely direct inhibition of AKT kinase activity, inhibition of AKT phosphorylation, activation of AKT dephosphorylation or prevention of AKT translocation to the plasma membrane^48^. Since tHGA was shown to inhibit AKT phosphorylation at Ser473 and Thr308, this finding points to the possibility that tHGA is causing inhibition of AKT activation.

There are basically two prominent cell regulatory events involved in AKT activation, which include phosphorylation of the AKT kinase active site and translocation of AKT kinase from the cytoplasm to the cell membrane via the PH domain of AKT. By using myr-AKT construct, the mechanism of tHGA action upon AKT was further explored. Myr-AKT is a constitutively active mutant of AKT. The myristoylation signal constructed in this AKT mutant allowed the recruitment of AKT to the plasma membrane without its PH domain, thus causing an increase of its basal level of phosphorylation even in the absence of any growth factor^49^. tHGA treatment caused a significant reduction in phosphorylation of AKT at both Ser473 and Thr308 in myr-AKT-expressing cells. This indicates that tHGA could possibly block the phosphorylation site of AKT that leads to AKT inactivation.

The effect of tHGA upon both cyclin D1 and p27^Kip1^ expression can be explained by the fact that AKT mediates cell cycle progression from the G_1_ to S phase through inactivation of GSK-beta, which leads to an increase in cyclin D1. Furthermore, inhibition of forkhead family transcripition factors and the tumor suppressor tuberin (TSC2) by active AKT results in the reduction of p27^Kip1 ^^50^. The protein stability of p27^Kip1^ is also reported to be mediated by AKT-dependent phosphorylation of Skp2^51^. Blocking AKT activity allows p27^Kip1^ to continuously inhibit the activity of CDKs, thus promoting cell cycle exit.

Apart from its role in cell cycle regulation, p27^Kip1^ is also involved in regulating cell migration via the Rho kinase pathway. Binding of p27^Kip1^ to RhoA interferes with the interaction between RhoA and its activator, thus inhibiting its activation^52^. It is possible that through inhibition of p27^Kip1^ reduction, tHGA could suppress ASM migration in response to growth factors through indirect effects upon the RhoA pathway. The inhibition of AKT by tHGA could also impact cellular migration through disruption of the PI3K pathway. PTEN, a tumour suppressor, was reported to inhibit human airway smooth muscle cell migration through AKT signaling pathway^53^. A study employing AKT null cells showed lack of polarization and reduced migration in response to cAMP^54^. Furthermore, AKT mediates the regulation of PAKa which is required for myosin assembly, thus in part, controlling cell polarity and chemotaxis^55^. A study demonstrated that tetrandrine (Tet), a calcium blocker isolated from *Stephania tetranda*, inhibited cell migration through its inhibition on AKT and JNK activation. In addition, their findings also revealed that AKT inhibitor (triciribine) and JNK inhibitor (SP600125) significantly attenuated cell migration^56^. These findings further imply that tHGA inhibition on AKT and JNK phosphorylation could be the reason that lead to the anti-migratory effect in hBSMCs.

In describing the mechanism involved in tHGA-induced cell cycle arrest, possible effects upon ASM apoptosis were evaluated in which tHGA was demonstrated to have no effect upon apoptosis. It has been reported that AKT upregulates expression of Bcl-2, an anti-apoptotic protein, through cAMP-response element-binding protein in the apoptotic pathway^57^. Hence one would suspect inhibition of AKT would lead to an increased rate of apoptosis. However, tHGA failed to cause any effect upon apoptosis in growth factor-induced hBSMCs. This could be due to the fact that Bcl-2 can be regulated through several other upstream activators, such as ERK and p38 of which were not targeted by tHGA, hence enabling normal regulation of Bcl-2 leading to prevention of apoptosis^58^. In addition, a study also suggested that ERK cooperate with other survival signaling pathways, such as PKC activation, to ensure that Bcl-2 function properly^59^. Furthermore, another study had demonstrated that AKT inhibition alone was not sufficient to induce apoptosis. Instead, inhibition of PI3K, which is the upstream activator of AKT, led to the decrease of the phosphorylation of AKT and ERK and subsequently causes apoptosis^60^. This further explains the possible reason that AKT inhibition by tHGA in this study did not lead to apoptosis induction.

Previous studies have reported that asthmatic ASM cells proliferate at a higher rate as compared to non-asthmatic ASM cells^61–63^. Proliferation of both asthmatic and non-asthmatic ASM were reported to be regulated via identical pathways, which includes the PI3K/AKT signalling pathway that was reported to be dominant in asthmatic ASM cells in response to strong mitogenic stimulation^62^. Furthermore, several studies had also demonstrated that the PI3K/AKT signalling pathway is integral in regulating proliferation of asthmatic ASM, in which proliferation is inhibited through inhibition of signalling molecules of the PI3K/AKT pathway^62,64,65^. It is highly likely that the inhibition demonstrated by tHGA in non-asthmatic ASM in this communication would occur through similar pathways in asthmatic ASM. Further work employing asthmatic ASM is envisaged in which the effect of tHGA upon established pathological changes can be assessed.

**Figure 7:**
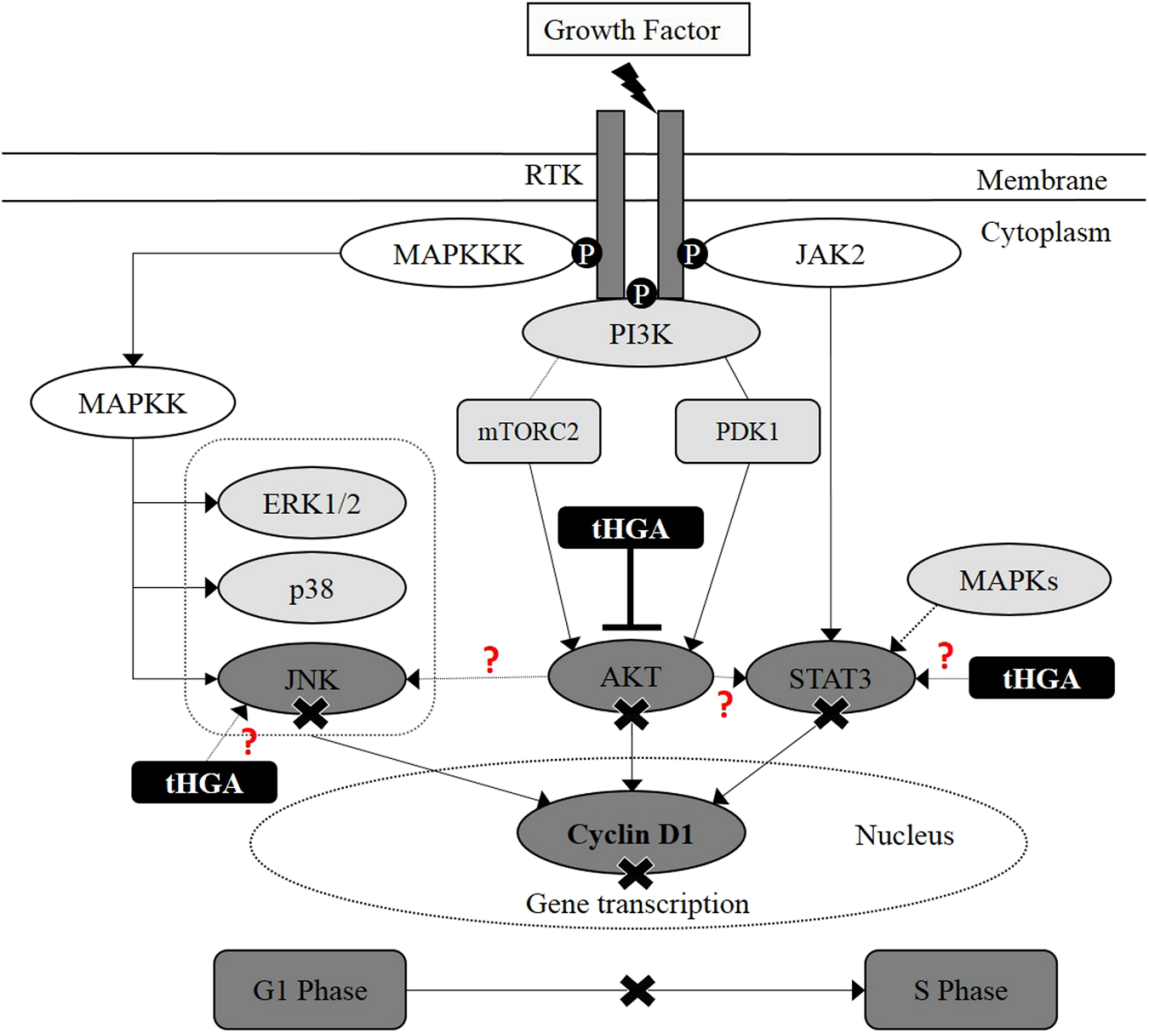
tHGA attenuates hBSMCs proliferation primarily via AKT inhibition. AKT was suggested to act as upstream activator for JNK and STAT3, thus making it more of the primary target of tHGA. However, further investigation is required as the possibility of multiple targets still exists. Further examinations should examine the role of JNK and STAT3 inhibition following tHGA treatment. tHGA inhibition on these few signaling molecules (AKT, JNK and STAT3) reduced cyclin D1 protein expression, thus causing cell cycle arrest at G_1_ phase.

Although the current study has determined that inhibition of AKT phosphorylation to be the major effector mechanism of tHGA upon bronchial smooth muscle proliferation and migration, the possibility of multiple targets still exists and would require further experimentation. Further investigations should examine the role of JNK and STAT3 inhibition and an understanding of alterations to cross-talk between these molecules and AKT following tHGA treatment. The role of tHGA on other upstream activators of JNK, including MEK4 and MEK7, as well as the activity of PI3K can be further examined. Nevertheless, in this communication, our findings showed that tHGA inhibits hBSMCs proliferation and migration without inducing apoptosis through inhibition of AKT, JNK and STAT3 signaling molecules. AKT was suggested to be the major molecular target of tHGA in hBSMCs upon growth factor stimulation (Fig. 7).

## Methods

### tHGA synthesis and preparation

tHGA was synthesized as reported previously^10^. tHGA stock (20 mM) was prepared by dissolving the compound in 100% DMSO. The final concentrations of DMSO in all assays was 0.1%.

### Culture and treatment of hBSMCs

hBSMCs were obtained from Lonza (Basel, Switzerland) and were maintained in smooth muscle basal medium (SmBM) supplemented with 5% (v/v) fetal bovine serum (FBS), 5 ng/ml insulin, 2 ng/ml human bFGF, 50 ng/ml human EGF, 50 µg/ml gentamicin and 50 ng/ml amphotericin at 37 °C with 5% CO_2_. Cells between passages 4 and 8 were used for all experiments. Prior to tHGA treatment on hBSMCs, hBSMCs (1.56 × 10^4^ cells/cm^2^) were seeded in complete media for 24 hours, followed by serum starvation for 24 hours with 0.1% FBS SmBM. Serum starved cells were then induced with growth factor-enriched media containing 5% (v/v) fetal bovine serum (FBS), 5 ng/ml insulin, 2 ng/ml human bFGF and 50 ng/ml human EGF and co-treated with/without tHGA at indicated time points. For assay controls, hBSMCs were treated with 10 µM forskolin, 5% DMSO or respective inhibitors.

### LDH cytotoxicity assay

hBSMCs were serum starved for 24 hours prior to induction with growth factor-enriched media and co-treatment with/without tHGA for 48 hours in 96-well culture plates with a seeding density of 5000 cells per well. The LDH cytotoxicity assay was performed according to the manufacturer’s protocol (Thermo Scientific, Rockford, USA). Non-toxic concentrations of tHGA were determined and used for all subsequent assays.

### BrdU proliferation assay

The BrdU cell proliferation assay was carried out according to the manufacturer’s instructions (Merck Milipore, Darmstadt, Germany). Briefly, hBSMCs were seeded at 5000 cells per well in 96-well culture plates and were serum starved for 24 hours prior to induction with growth factor-enriched media and co-treatment with/without tHGA for 48 hours. Cell proliferation was determined through the measurement of the BrdU incorporation during DNA synthesis, thus BrdU reagent was added 24 hours prior to the end of treatment. Anti-BrdU monoclonal antibody was then added to detect the BrdU-incorporated cells, followed by addition of IgG-peroxidase conjugated secondary antibody, substrate and stop solution. Lastly, the BrdU-incorporated cells were measure through colorimetric detection at dual wavelength of 450/550 nm.

### Reverse transcription polymerase chain reaction (RT-PCR)

Serum starved hBSMCs were induced with growth factor and co-treated with/without tHGA for 48 hours in T25 flasks. RNA was extracted with the RNeasy Mini kit (Qiagen, Hilden, Germany) and RT-PCR was carried out using Qiagen OneStep RT-PCR kit (Qiagen, Hilden, Germany). Primers used for the Ki-67 gene were: forward, 5′-CTA CTC CAA AGA AGC CTG TG-3′; reverse, 5′-AAT GAA GTT GTT GAG CAC TCT G-3′ while for the glyceraldehyde 3-phosphate dehydrogenase (GAPDH) gene primers used were: forward, 5′-GCC GCA TCT TCT TTT GCG TC-3′; reverse, 5′-TCG CCC CAC TTG ATT TTG GA-3′. The thermal cycler (Eppendorf, Hamburg, Germany) was programmed for 30 minutes reverse transcription at 50 °C followed by 30 minutes PCR activation step at 95 °C and 32 cycles with 30 seconds denaturation at 94 °C, 30 seconds annealing at 58 °C and 1 minute elongation at 72 °C. The elongation step for the final cycle was extended for another 10 minutes at 72 °C. The PCR products were analyzed on a 2% agarose gel with ethidium bromide staining. Densitometry of the PCR bands was quantitated by using Image J Image Processing Software (NIH, USA) and normalized to GAPDH.

### Cell cycle analysis

Serum starved hBSMCs were induced with growth factor-enriched medium and co-treated with/without tHGA for 48 hours in T25 flasks. Cells were trypsinized and washed twice with phosphate-buffered saline (PBS), followed by fixing and permeabilization with 70% ethanol for 30 minutes at 4 °C. Cells were then spun down and incubated with RNase A (100 µg/ml) for 5 minutes. Next, propidium iodide (50 µg/ml) was added and cells incubated for 15 minutes at room temperature in the dark. Stained cells (a total of at least 10000 events) were analyzed with a BD LSR Fortessa flow cytometer (BD, San Diego, USA).

### Flow cytometric analysis of apoptosis

Serum starved hBSMCs were induced with growth factor-enriched medium and co-treated with/without tHGA for 48 hours in T25 flasks. Cells were trypsinized and washed twice with PBS prior to annexin V/propidium iodide staining. The annexin V/propidium iodide assay was performed using a FITC annexin V apoptosis detection kit according to the manufacturer’s instructions (BD Pharmingen, San Diego, USA). Stained cells (a total of at least 10000 events) were analyzed with a BD LSR Fortessa flow cytometer (BD, San Diego, USA).

### Cell migration assays

#### Scratch assay

hBSMCs were seeded in 12-well culture plates, cultured until confluent and serum-starved for 24 hours. Linear scratches were made on cell monolayers with sterile pipette tips and cell debris was removed by washing with PBS. Serum starved hBSMCs were induced with growth factor-enriched medium and co-treated with/without tHGA. Scratched areas were marked with a permanent marker and images were captured with Dino-Eye eyepiece camera (Dino-Lite Digital Microscope, New Taipei City, Taiwan) attached to an inverted light microscope (Leica Microsystem, Wetzlar, Germany) at 0, 24 and 48 hours post-treatment. The total number of migrated cells per area was ccounted based on 3 representative images captured for each treatment condition^66^.

#### Transwell migration assay

hBSMCs were serum-starved for 24 hours prior to seeding into 8 µM pore membrane inserts (transparent PET membrane) (BD Biosciences, New Jersey, USA) (5000 cells per well), pre-coated with 1.5 mg/mL type 1 rat-tail collagen (Corning, Cambridge, USA). Cells were maintained in serum-free media with or without tHGA/forskolin. Growth factor-enriched media was added into transwell lower chambers (24 well-plate) with or without tHGA/forskolin. Lower chambers containing media with 0.1% FBS were included to serve as negative controls. Following a 6 hour incubation period, non-migrated cells (upper chamber) were removed with a sterile cotton swab^67^. Media was removed from the lower chamber followed by addition of 500 µL of fresh media and 100 µl of MTS solution and incubated for 4 hours followed by measurement of absorbance at 490 nm through microplate reader (Molecular Devices, California, USA)^68^.

### Western blot analysis

Serum starved hBSMCs were induced with growth factors and co-treated with/without tHGA in T75 flasks at indicated time. Following treatment, cells were lysed with RIPA lysis buffer containing 150 mM NaCl, 50 mM Tris HCL, 0.1% SDS, 1% Triton X, 0.5% sodium deoxycholate, protease inhibitor (1:100) and phosphatase inhibitor (1:100). Then, lysates were shaken on ice for 15 minutes prior to centrifugation at 14,000 × g for 10 minutes. Protein concentration was estimated using a BCA protein assay kit (Novagen, Darmstadt, Germany). Equal amount of whole cell lysates (10 µg/lane) were separated by 8–12% SDS-PAGE electrophoresis for 1–2 hours at 100 V and transferred onto 0.2 µm polyvinylidene difluoride (PVDF) membranes using a semi-dry transfer system (Bio-Rad Laboratories, California, USA) at 0.25 mA for 8–10 minutes. Membranes were blocked with 5% bovine serum albumin (BSA) for 1 hour followed by an overnight incubation with primary antibodies. Membranes were then washed thrice with tris-buffered saline/Tween 20 (TBST) followed by incubation with secondary antibodies for 1 hour. Antibodies were prepared in 5% BSA according to the recommended dilution (see Supplementary Materials) and were used before in other studies. Bands were visualized by enhanced chemiluminescence (ECL) and imaged on a Vilber Fusion gel documentation system (Marne-la-Vallée, Frence). Membrane were stripped and reprobed as required. Densitometry of bands was conducted with Image J Image Processing Software (NIH, USA) and normalized to β-actin.

### Kinase activity assay

#### PDK1 kinase activity

Pierce crosslink magnetic IP/Co-IP kit (Thermo Scientific, Rockford, USA) was used for immunoprecipitation (IP) according to the manufacturer’s instructions. An anti-PDK1 antibody (Cell Signaling Technology, Boston, United States) was used to extract cellular PDK1 from the total protein lysates. Immunoprecipitates were then incubated with PDK1 kinase buffer containing 1× kinase buffer, 200 µM adenosine triphosphate (ATP) (Cell Signaling Technology, Boston, United States) and 500 ng of AKT substrate (Merck Milipore, Darmstadt, Germany) at 30 °C for 30 minutes. Kinase activity was stopped with 1× Laemmli loading buffer. Proteins were separated on 8% SDS-PAGE for 1–2 hour at 100 V and transferred onto PVDF membranes using a semi-dry transfer system (Bio-Rad Laboratories, California, USA) at 0.25 mA for 10 minutes. Membranes were then blocked with 5% bovine serum albumin (BSA) for 1 hour followed by an overnight incubation with primary antibodies against phospho-AKT and AKT (Cell Signaling Technology, Boston, United States). Membranes were then washed thrice with tris-buffered saline/Tween 20 (TBST) followed by incubation with secondary antibodies for 1 hour. Antibodies were prepared in 5% BSA according to the recommended dilution (see Supplementary Materials). Bands were visualized by enhanced chemiluminescence (ECL) and imaged on a Vilber Fusion gel documentation system (Marne-la-Vallée, Frence). Densitometry of bands was conducted with Image J Image Processing Software (NIH, USA).

#### mTORC2 kinase activity

Pierce crosslink magnetic IP/Co-IP kit (Thermo Scientific, Rockford, USA) was used for IP according to the manufacturer’s instructions. However, the IP lysis buffer was replaced with CHAPS lysis buffer (pH 7.4) containing 40 mM HEPES, 120 mM NaCl, 2 mM EDTA, 0.3% CHAPS, 10 mM pyrophosphate, 10 mM glycerophosphate, protein inhibitor cocktail (1:100) and phosphatase inhibitor cocktail (1:100). An anti-rictor antibody (Cell Signaling Technology, Boston, United States) was used to co-immunoprecipitate the mTORC2 complex from total cellular protein. An anti-mTOR antibody (Cell Signaling Technology, Boston, United States) was used to detect the mTORC2 complex via western blot analysis. Protein lysate was separated on 8% SDS-PAGE and immunoblotted with antibodies against Rictor and mTOR (Cell Signaling Technology, Boston, United States) and transferred onto PVDF membrane through wet transfer system (Bio-Rad Laboratories, California, USA) (1.5 hours, 0.35 mA). Antibodies were prepared in 5% BSA according to the recommended dilution (see Supplementary Materials). Membranes were then blocked with 5% bovine serum albumin (BSA) for 1 hour followed by an overnight incubation with primary antibodies against mTOR and Rictor (Cell Signaling Technology, Boston, United States). Bands were visualized by enhanced chemiluminescence (ECL) and imaged on a Vilber Fusion gel documentation system (Marne-la-Vallée, Frence). Membrane were stripped and reprobed as required. Densitometry of bands was conducted with Image J Image Processing Software (NIH, USA).

Immunoprecipitates were incubated in mTORC2 kinase buffer containing 25 mM HEPES, 100 mM potassium acetate and 1 mM MgCl at 30 °C for 30 minutes. Kinase activity was stopped with 1× Laemmli loading buffer and proteins were separated on 10% SDS-PAGE and immunoblotted with antibodies against phospho-AKT (Ser473) and AKT (Cell Signaling Technology, Boston, United States). Antibodies were prepared in 5% BSA according to the recommended dilution (see Supplementary Materials). Bands were visualized by enhanced chemiluminescence (ECL) and imaged on a Vilber Fusion gel documentation system (Marne-la-Vallée, Frence). Membrane were stripped and reprobed as required. Densitometry of bands was conducted with Image J Image Processing Software (NIH, USA).

### Transfection of myr-AKT and pECE

The plasmid myrAkt delta4–129 (myr-AKT) was a gift from Richard Roth^49^ (Addgene plasmid # 10841) while the empty vector (pECE) was a gift from William Rutter^69^ (Addgene plasmid # 26453). Plasmids were received as bacteria in agar stabs. Bacterial colony isolation was carried out by streak plating on Luria Bertani (LB) agar for 16 hours followed by inoculation into LB broth and 16 hour incubation at 37 °C on an orbital shaker (200 rpm) (Thermo Scientific, Massachusetts, United States). Plasmids were extracted with EndoFree® Plasmid Maxi Kit (Qiagen, Hilden, Germany) according to the manufacturer’s instructions. Transient transfection of hBSMCs with myr-AKT or pECE (1.5 µg) was performed via Nucleofection^TM^, an electroporative technique developed by Lonza Group Ltd. (Basel, Switzerland). Prior to this, transfection condition was optimized using the pmaxGFP^TM^ vector, provided with the nucleofector kit, according to the manufacturer’s instruction to determine the transfection efficiency and cell viability post transfection. Optimization of transfection condition was performed with 6 different Nucleofector^TM^ programs plus 1 negative control (no program). Briefly, hBSMCs (1 × 10^6^ cells) were resuspended in 100 µL of 4D-Nucleofector^TM^ mastermix (Lonza, Basel, Switzerland) and run in a 4D-Nucleofector^TM^ System (Lonza, Basel, Switzerland) for each transfection condition. Cells were then seeded in T25 flask (cell viability) and Falcon CultureSlides (8-well) (transfection efficiency) (BD Biosciences, New Jersey, USA) with complete media for 48 hours. hBSMCs in Falcon CultureSlides was fixed with 4% paraformaldehyde followed by Hoechst 33342 DNA staining. GFP-expressing hBSMCs were visualized under fluorescence microscope at excitation 488 nm and emission 509 nm while Hoechst stained cells were detected at excitation 352 nm and emission 455 nm. Transfection efficiency was determined by counting GFP-expressing cells over total cells. hBSMCs viability post transfection was determined through trypan blue exclusion method. The optimum Nucleofector^TM^ program was determined and was used for pECE or Myr-AKT transfection in hBSMCs. Myr-AKT expressing hBSMCs were treated with/without 20 µM tHGA for 1 hour. Following treatment, cells were lysed with RIPA lysis buffer and protein lysate was then separated with 10% SDS-PAGE (semi-dry transfer system, 0.25 mA, 10 minutes) and immunoblotted with antibodies against HA-tag, phospho-AKT (Ser473), phospho-AKT (Thr308) and β-actin. Antibodies were prepared in 5% BSA according to the recommended dilution (see Supplementary Materials). Bands were visualized by enhanced chemiluminescence (ECL) and imaged on a Vilber Fusion gel documentation system (Marne-la-Vallée, Frence). Membrane were stripped and reprobed as required. Densitometry of bands was conducted with Image J Image Processing Software (NIH, USA).

### Statistical analysis

Data are expressed as means ± SEM and analyzed by one way ANOVA followed by Dunnett’s post hoc test (IBM SPSS Statistics, Version 20.0) by comparison to growth-factor-induced control. A *P*-value < 0.05 was considered to be statistically significant.

## Electronic supplementary material

Supplementary Information

## Data Availability

All data generated or analysed during this study are included in this published article (and its Supplementary Information file).
