# Supplementary material for: The geranyl acetophenone tHGA attenuates human bronchial smooth muscle proliferation via inhibition of AKT phosphorylation
Source: Sci Rep. 2018 Nov 9;8:16640. doi: 10.1038/s41598-018-34847-0 (PMC6226528; doi:10.1038/s41598-018-34847-0)
Supplement: Supplementary file 1 — Supplementary Information [file 41598_2018_34847_MOESM1_ESM.doc]

**The geranyl acetophenone tHGA attenuates human bronchial smooth muscle proliferation via inhibition of AKT phosphorylation**

Hui Min Yap^1^, Yu Zhao Lee^1^, Hanis Hazeera Harith^1^, Chau Ling Tham^1^, Manraj Singh Cheema^1^, Khozirah Shaari^2^, and Daud Israf Ali^1*^

^1^Department of Biomedical Science, Faculty of Medicine and Health Sciences, Universiti Putra Malaysia, 43400 Serdang, Selangor, Malaysia

^2^Institute of Bioscience, Universiti Putra Malaysia, 43400 Serdang, Selangor, Malaysia

^*^[daudaia@upm.edu.my](mailto:daudaia@upm.edu.my)

**Supplementary Materials**

Reagents and antibodies

Antibodies against cyclin D1 (1:1000; #2922), p27^KIP1^ (1:1000; #2552), phospho-JAK2 (Tyr1007/1008) (1:1000; #3771), JAK2 (1:1000; #3229), phospho-STAT3 (Tyr705) (1:1000; #9131), STAT3 (1:2000; #4904), phospho-p44/42 MAPK (Erk1/2) (Thr202/Tyr204) (1:2000; #9101), p44/42 MAPK (Erk1/2) (1:2000; #9102), phospho-SAPK/JNK (Thr183/Tyr185) (1:1000; 9251), SAPK/JNK (1:1000; #9252), phospho-p38 MAPK (Thr180/Tyr182) (1:1000; #9211), p38 MAPK (1:1000, #9212), phospho-PI3 Kinase p85 (Tyr458) (1:1000; #4228), PI3 Kinase p85 (1:1000; #4292), phospho-PDK1 (Ser241) (1:1000; #3061), PDK1 (1:1000; #3062), phospho-Akt (Ser473) (1:1000; #9271), phospho-Akt (Thr308) (1:1000; #4056), Akt (1:1000; #9272), Rictor (1:1000; #2114), mTOR (1:1000; #2972) and HA-tag (1:1000; #3724) were purchased from Cell Signaling Technology (Boston, United States). Goat anti-rabbit IgG-HRP secondary antibody (1:5000; sc-2004), β-actin antibody (1:10000; sc-47778) and AG490 (JAK2 inhibitor) (CAS 133550-30-8) were purchased from Santa Cruz (Texas, United States). Inhibitors include SB202190 (ab120638), SP600125 (ab120065), PD98059 (ab120234), triciribine (ab120936), S3I-201 (ab141434) and BX-795 (ab142016) were purchased from Abcam (Cambridge, United States) while LY294002 (#9901) was purchased from Cell Signaling Technology (Boston, United States). Forskolin (51328-34) was obtained from Nacalai Tesque (Kyoto, Japan). Low molecular weight DNA ladder was purchased from New England BioLabs (Massachusetts, United States) while BLUeye prestained protein ladder was obtained from Genedirex (United States).

**Supplementary Figure S1**


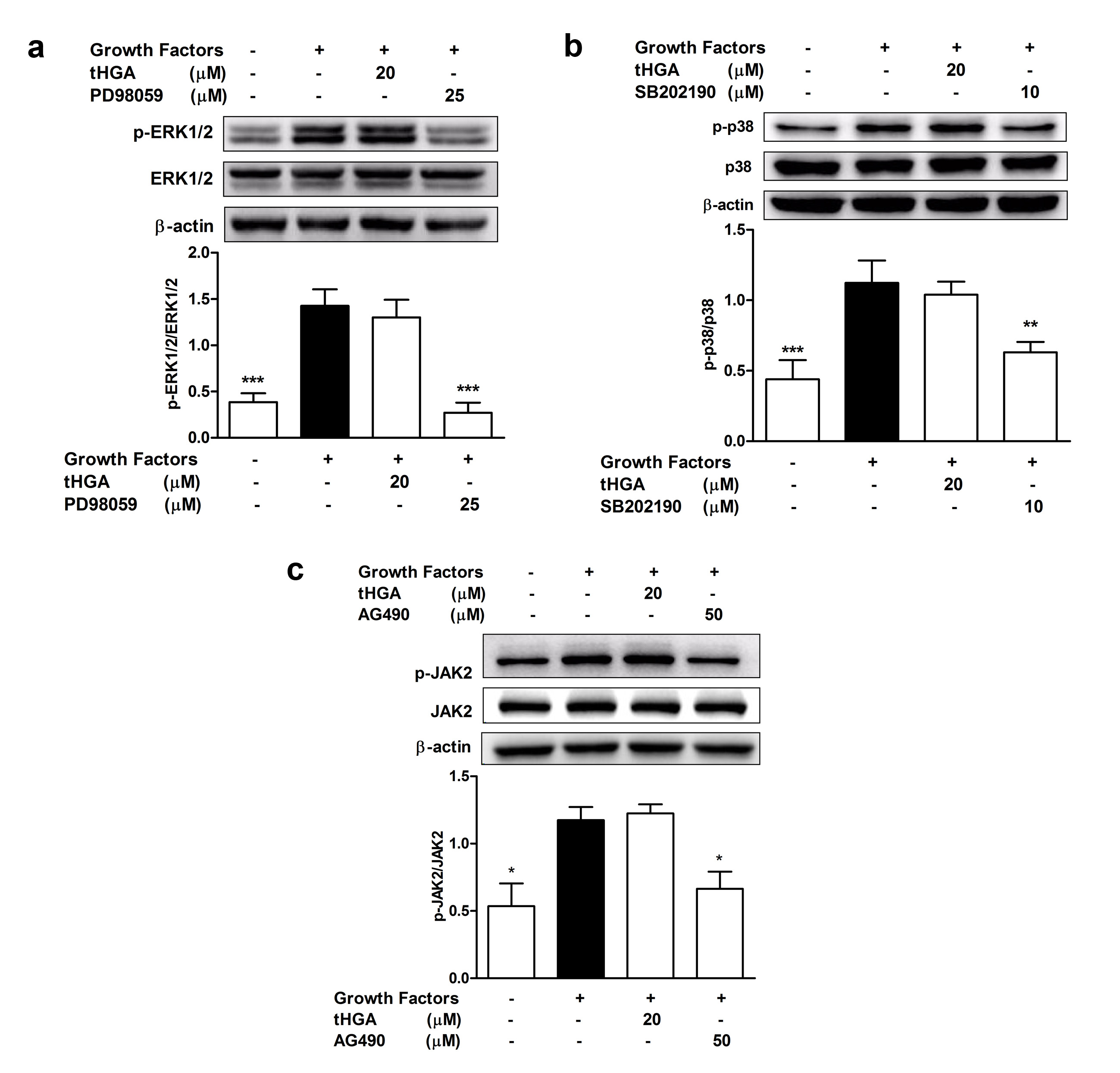


**Figure S1.** Effects of tHGA on the phosphorylation of ERK1/2, p38 and JAK2 in growth factor-induced hBSMCs. hBSMCs were serum-starved overnight before co-treatment with growth factors and tHGA or respective inhibitors (PD98059, SB202190 or AG490) for 1 hour. Proteins were immunoblotted with respective antibodies and the protein bands were quantified by densitometry. Results of (**a**) phospho-ERK1/2, (**b**) phospho-p38 and (**c**) phospho-JAK2 were presented after normalization with total ERK1/2, p38 and JAK2 respectively. Representative cropped blots are presented (full-length blots are presented in Supplementary Fig. S4 online). Results are presented as mean ± SEM of 3 independent experiments. **P*<0.05, ***P*<0.01 and ****P*<0.001, significantly different from growth factor induced-hBSMCs.

**Supplementary Figure S2**


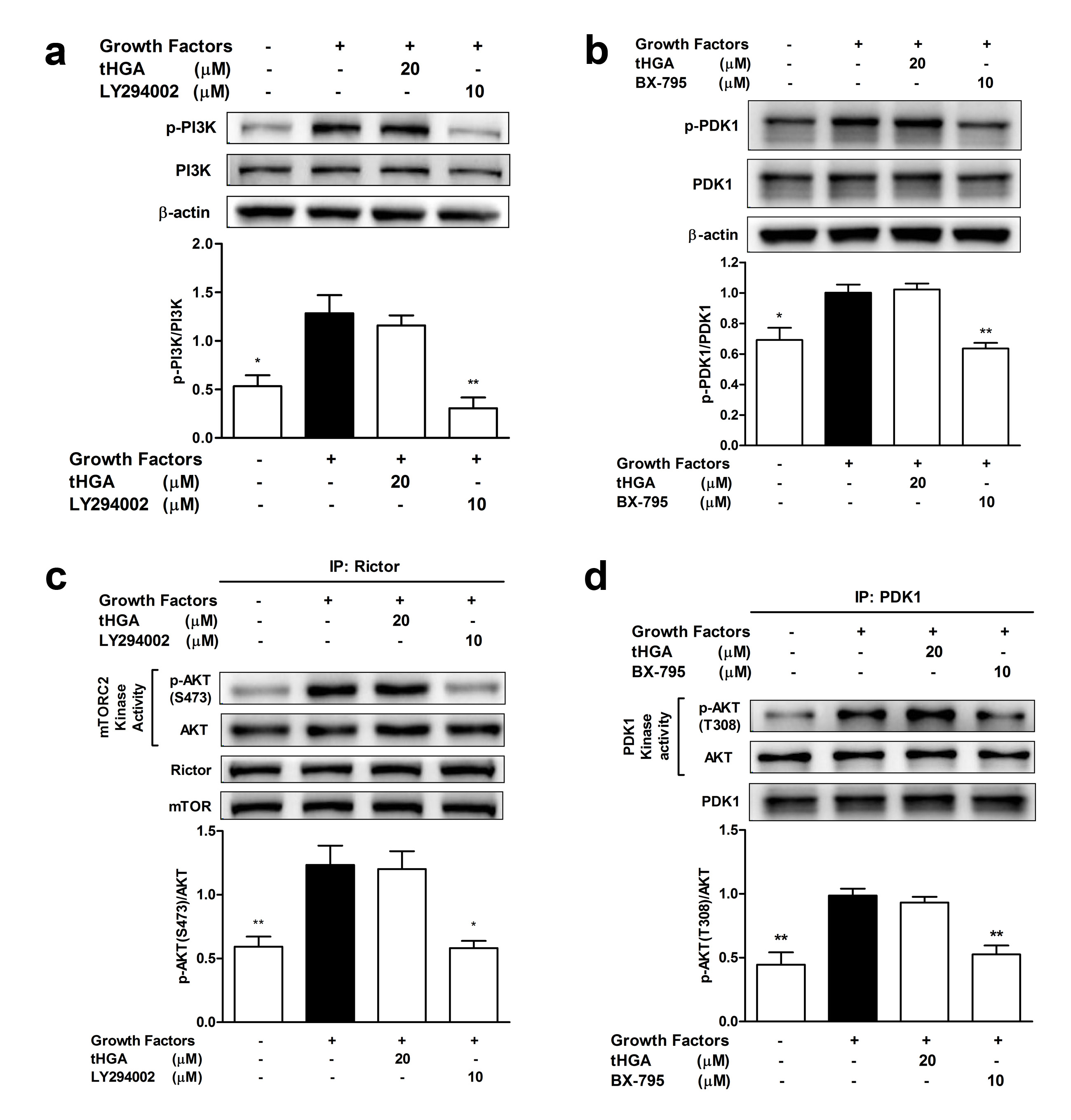


**Figure S2.** Effects of tHGA on the upstream activators of AKT in growth factor-induced hBSMCs. hBSMCs were serum-starved overnight before co-treatment with growth factors and tHGA or respective inhibitors (LY294002, or BX-795) for 1 hour. Phosphorylation of (**a**) PI3K and (**b**) PDK1 in growth factor-induced hBSMCs upon tHGA treatment were assessed through immunoblotting. Proteins were immunoblotted with respective antibodies and the protein bands were quantified by densitometry. Results of (**a**) phospho-PI3K and (**b**) phospho-PDK1 were presented after normalization with respective total proteins. Kinase activity of (**c**) mTORC2 and (**d**) PDK in growth factor-induced hBSMCs upon tHGA treatment were assessed through immunoprecipitation and immunoblotting. Proteins were immunoprecipitated with (**c**) anti-phospho-Rictor or (**d**) anti-PDK1 antibodies before subjected to kinase assay. Kinase assay sample was then immunoblotted with respective antibodies. Representative cropped blots are presented (full-length blots are presented in Supplementary Fig. S4 online). Results are presented as mean ± SEM of 3 independent experiments. **P*<0.05, ***P*<0.01 and ****P*<0.001, significantly different from growth factor induced-hBSMCs.

**Supplementary Figure S3**

Full-length Agarose Gel Image


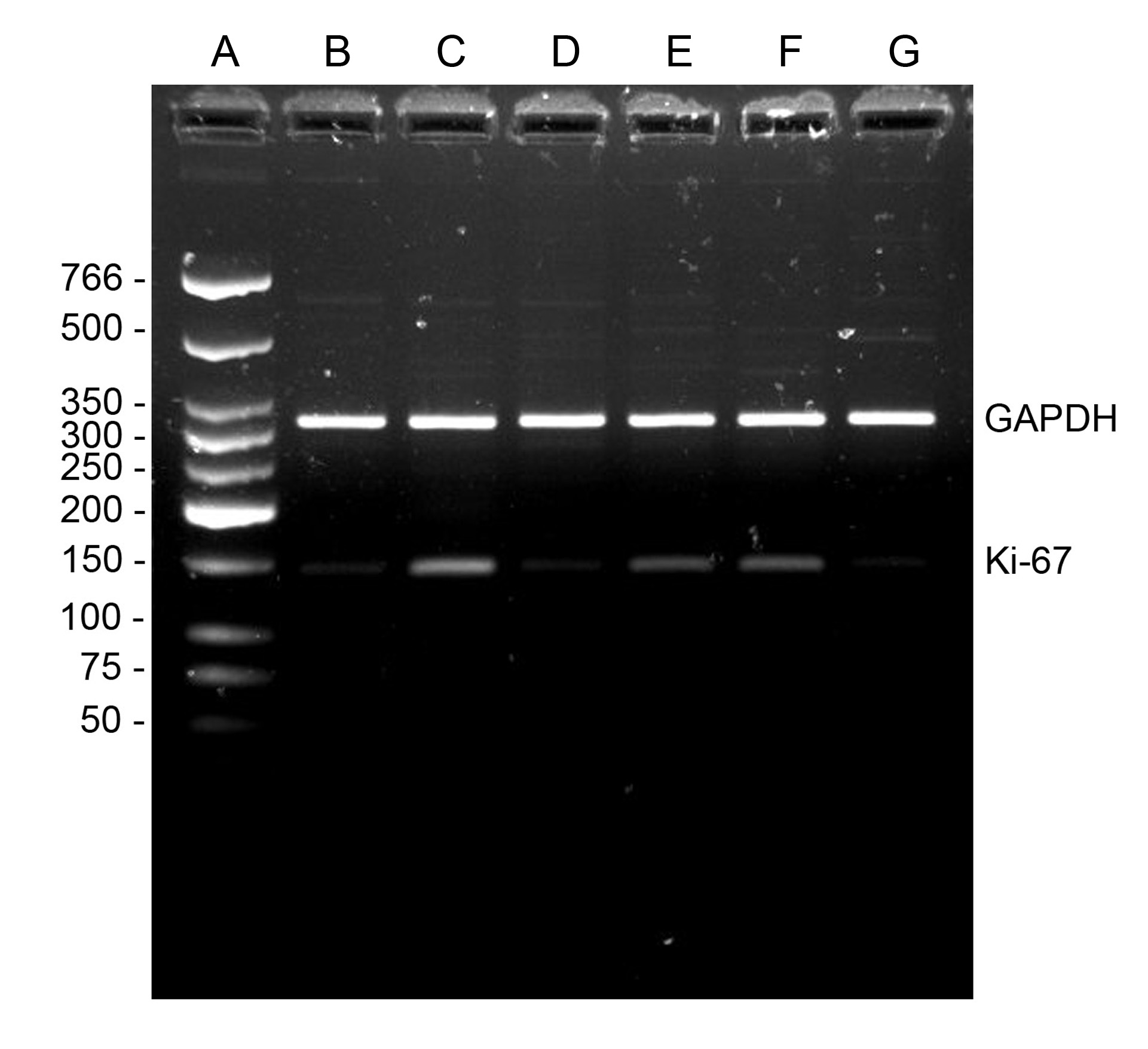


Representative Agarose Gel Image for Figure 1c

A – Low molecular weight DNA ladder

B – Untreated hBSMCs

C – hBSMCs induced with growth factor

D – hBSMCs induced with growth factor + 20 µM tHGA

E – hBSMCs induced with growth factor + 10 µM tHGA

F – hBSMCs induced with growth factor + 5 µM tHGA

G – hBSMCs induced with growth factor + 10 µM forskolin

**Supplementary Figure S4**

Full-length Western Blot Images


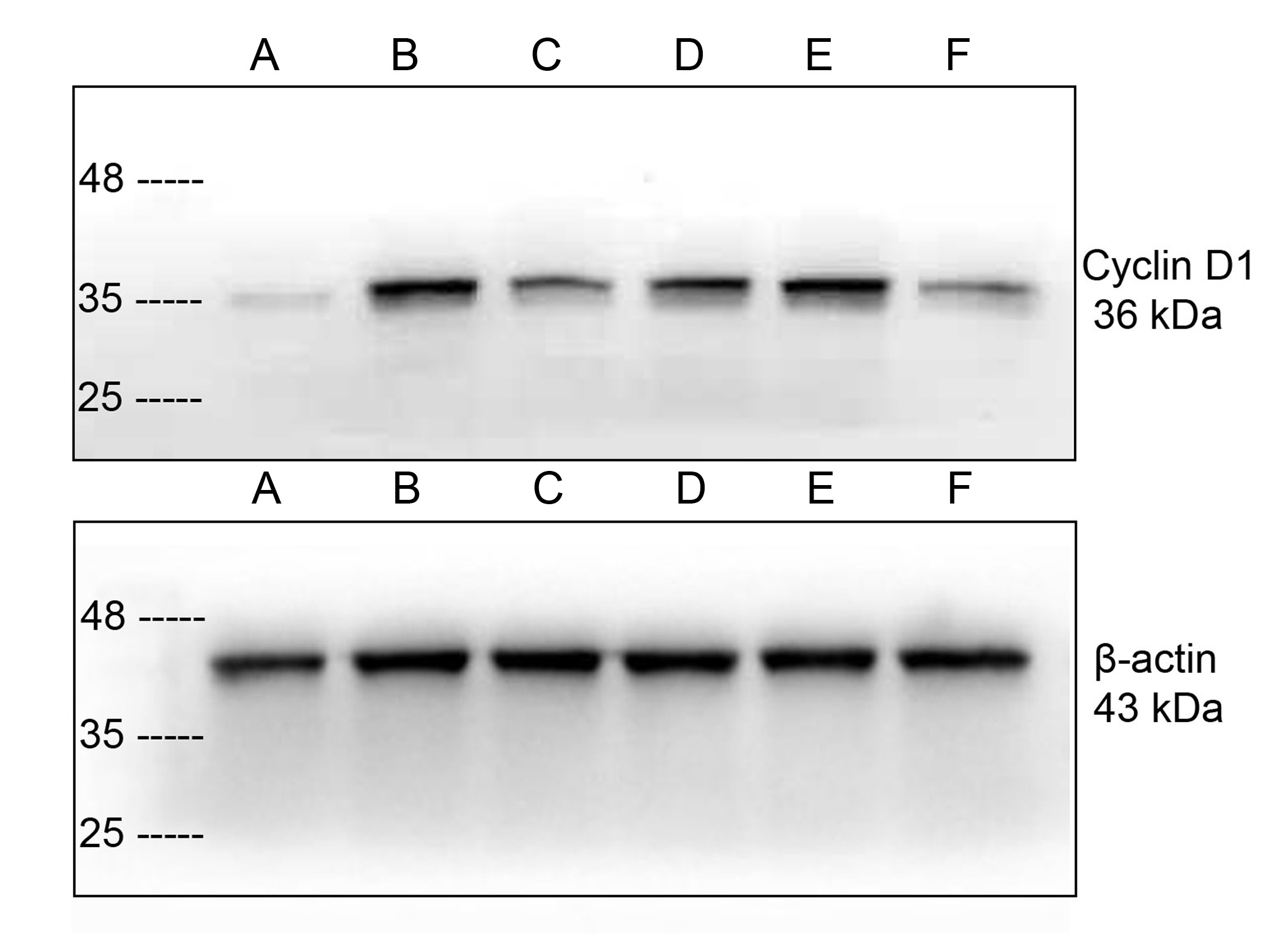


Representative Blot Images for Figure 4a

A – Untreated hBSMCs

B – hBSMCs induced with growth factor

C – hBSMCs induced with growth factor + 20 µM tHGA

D – hBSMCs induced with growth factor + 10 µM tHGA

E – hBSMCs induced with growth factor + 5 µM tHGA

F – hBSMCs induced with growth factor + 10 µM forskolin


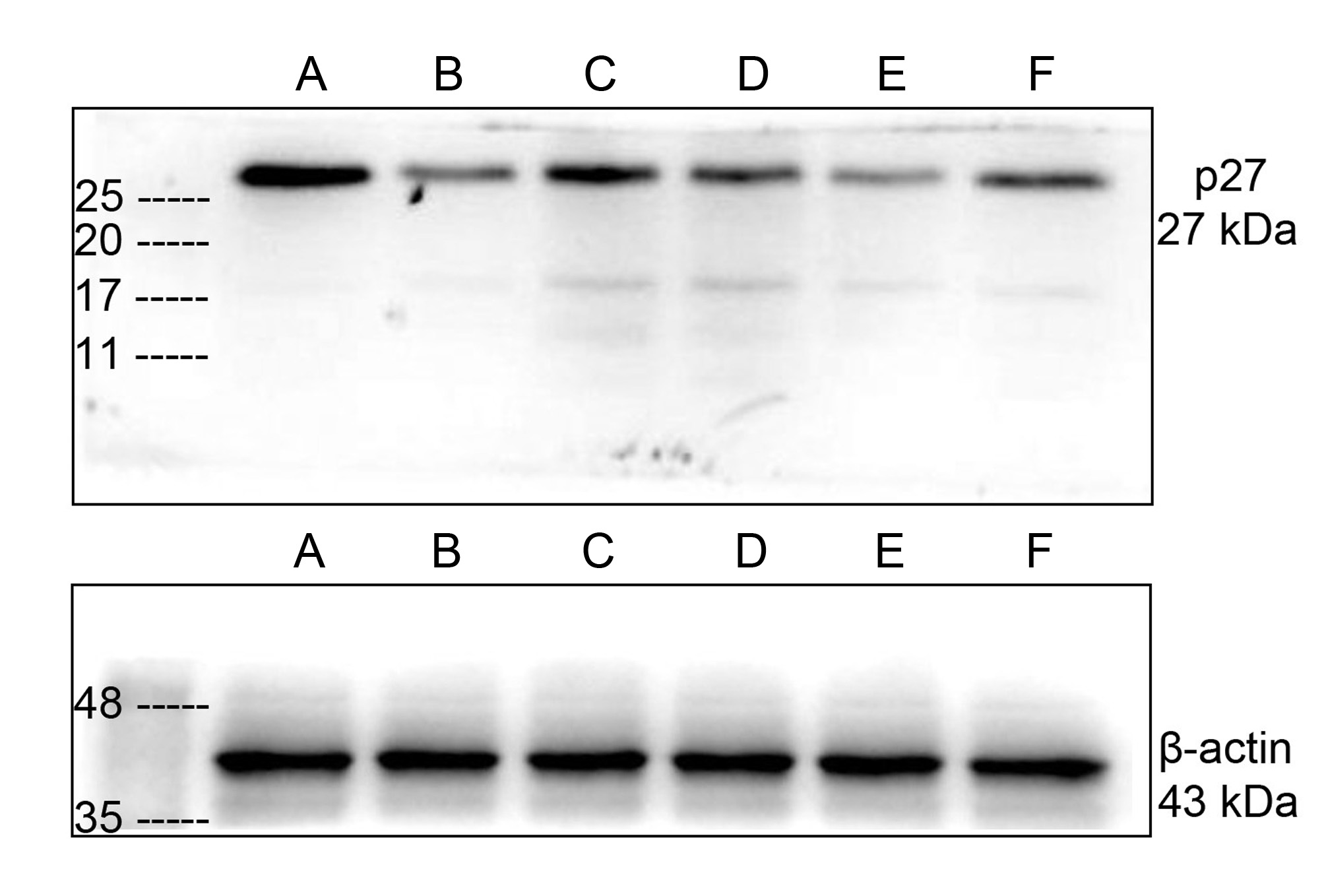


Representative Blot Images for Figure 4b

A – Untreated hBSMCs

B – hBSMCs induced with growth factor

C – hBSMCs induced with growth factor + 20 µM tHGA

D – hBSMCs induced with growth factor + 10 µM tHGA

E – hBSMCs induced with growth factor + 5 µM tHGA

F – hBSMCs induced with growth factor + 10 µM forskolin


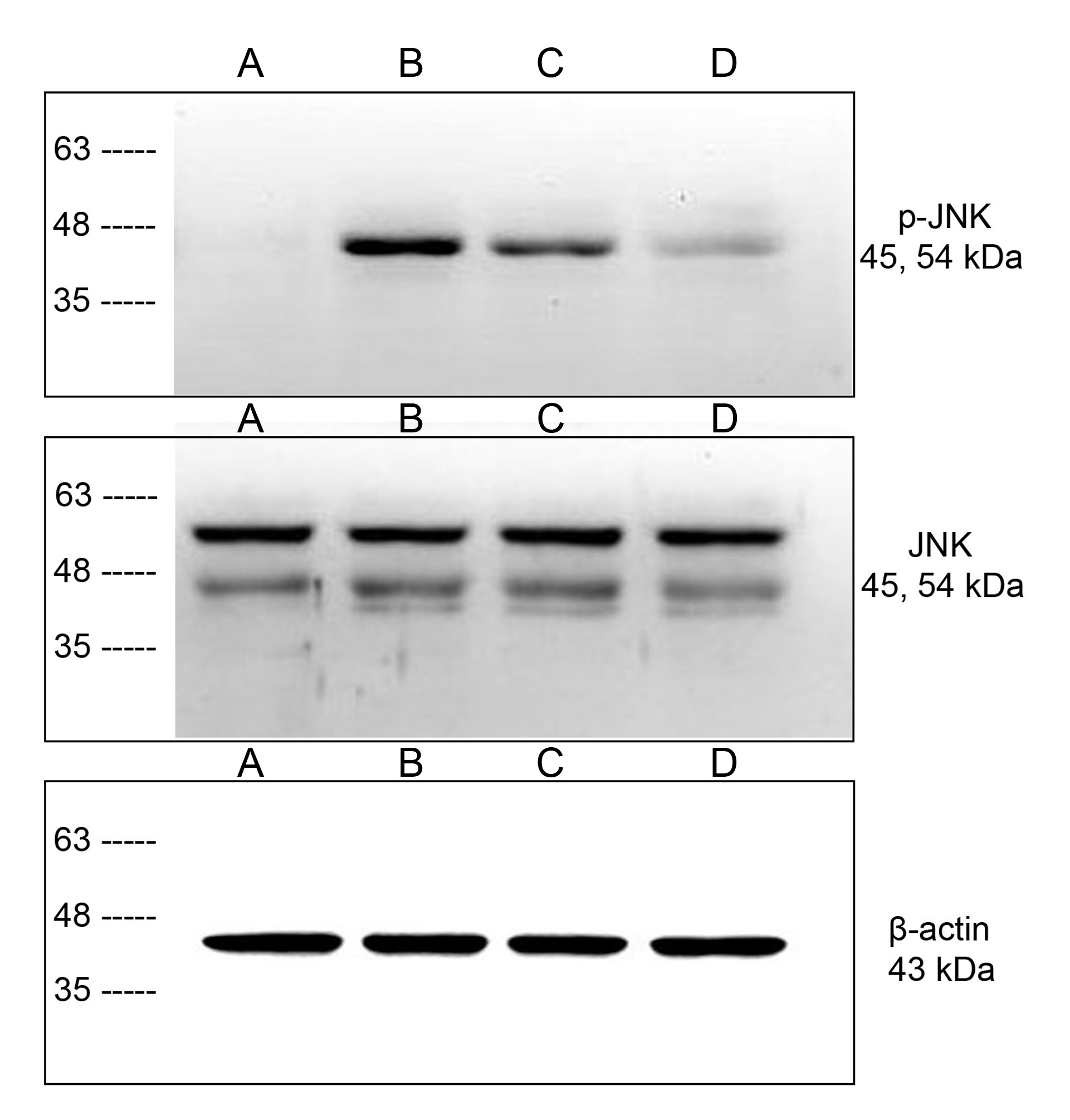


Representative Blot Images for Figure 4c

A – Untreated hBSMCs

B – hBSMCs induced with growth factor

C – hBSMCs induced with growth factor + 20 µM tHGA

D – hBSMCs induced with growth factor + 25 µM SP600125


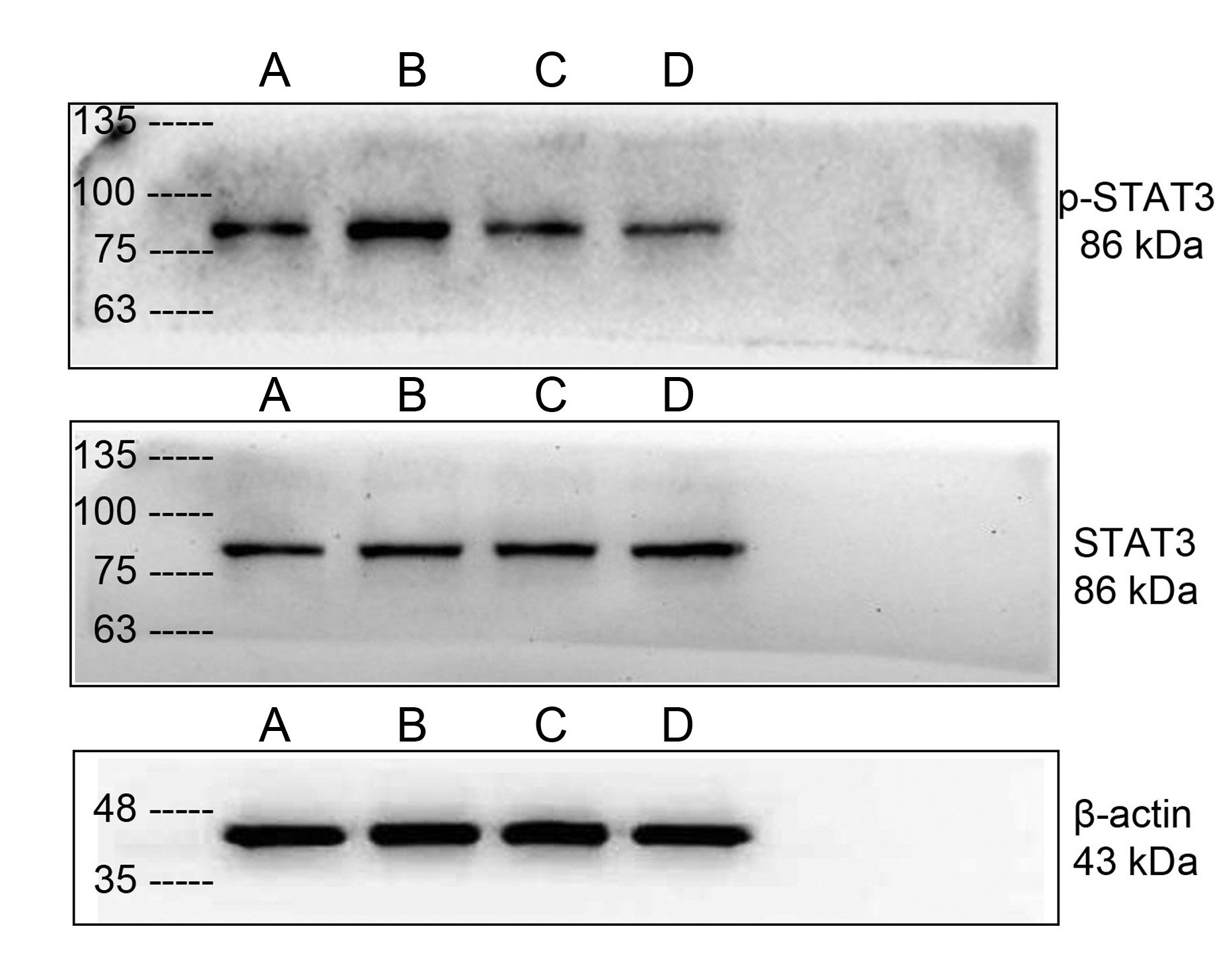


Representative Blot Images for Figure 4d

A – Untreated hBSMCs

B – hBSMCs induced with growth factor

C – hBSMCs induced with growth factor + 20 µM tHGA

D – hBSMCs induced with growth factor + 50 µM S3I-021


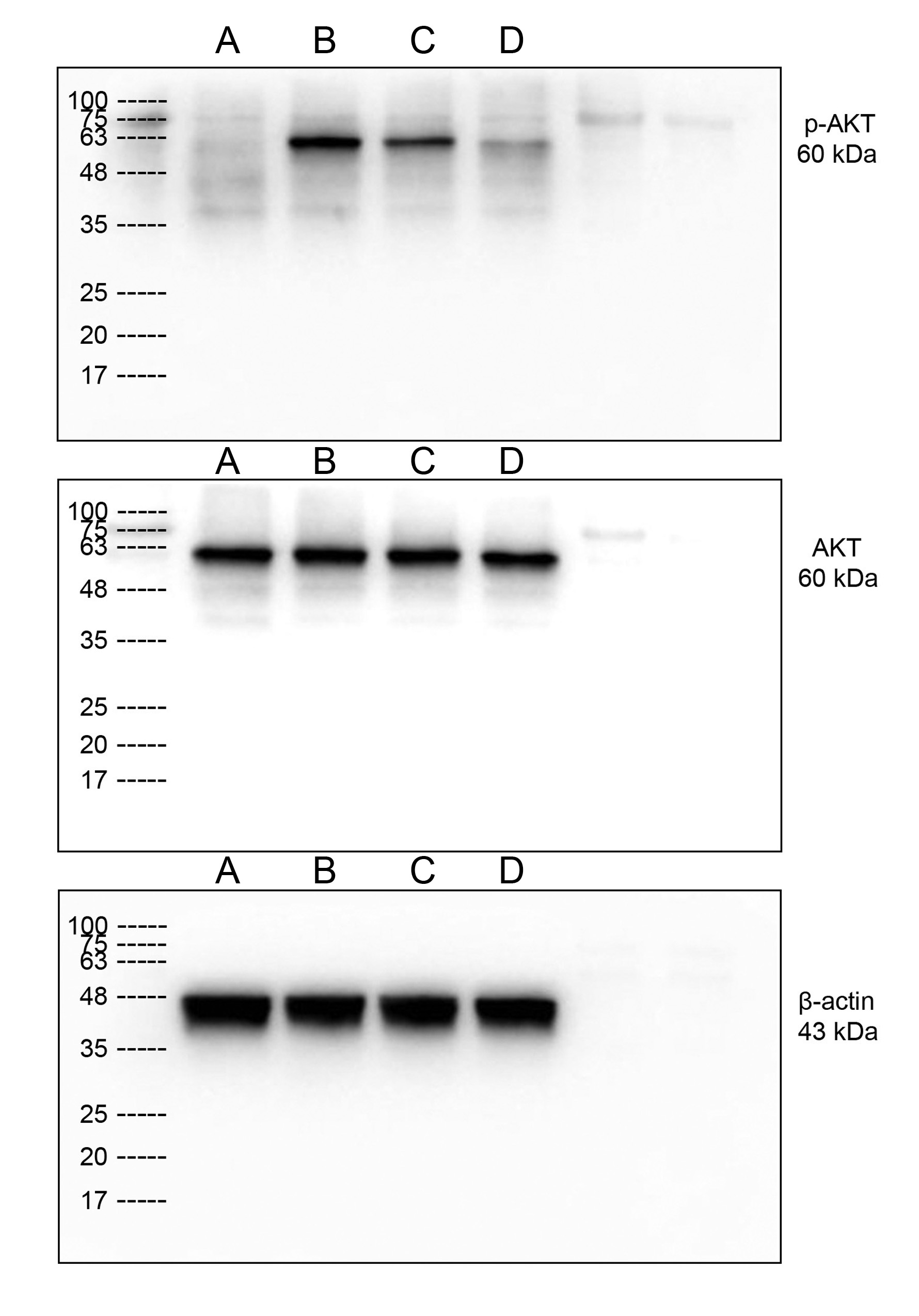


Representative Blot Images for Figure 5a

A – Untreated hBSMCs

B – hBSMCs induced with growth factor

C – hBSMCs induced with growth factor + 20 µM tHGA

D – hBSMCs induced with growth factor + 10 µM Triciribine


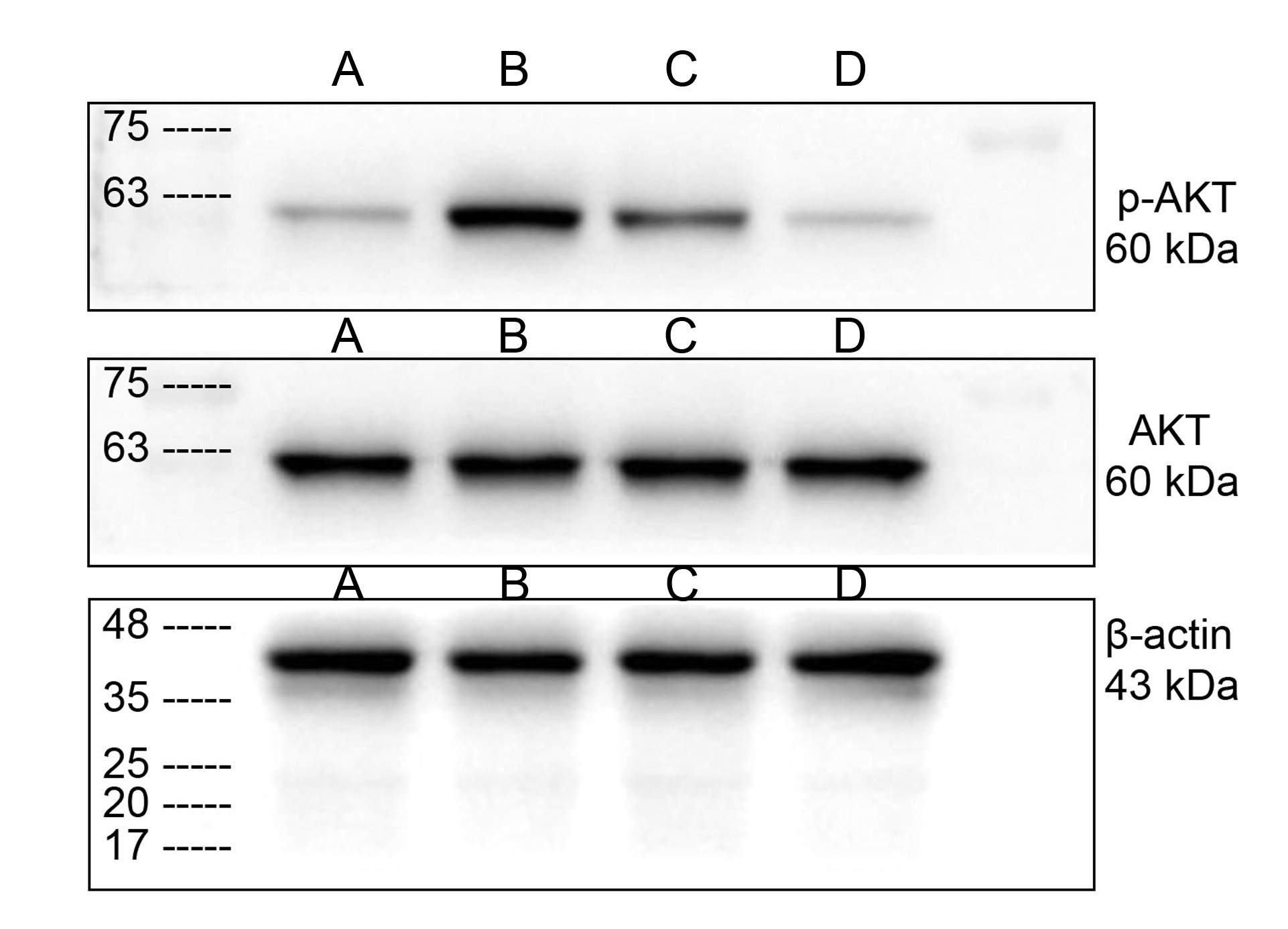


Representative Blot Images for Figure 5b

A – Untreated hBSMCs

B – hBSMCs induced with growth factor

C – hBSMCs induced with growth factor + 20 µM tHGA

D – hBSMCs induced with growth factor + 10 µM Triciribine


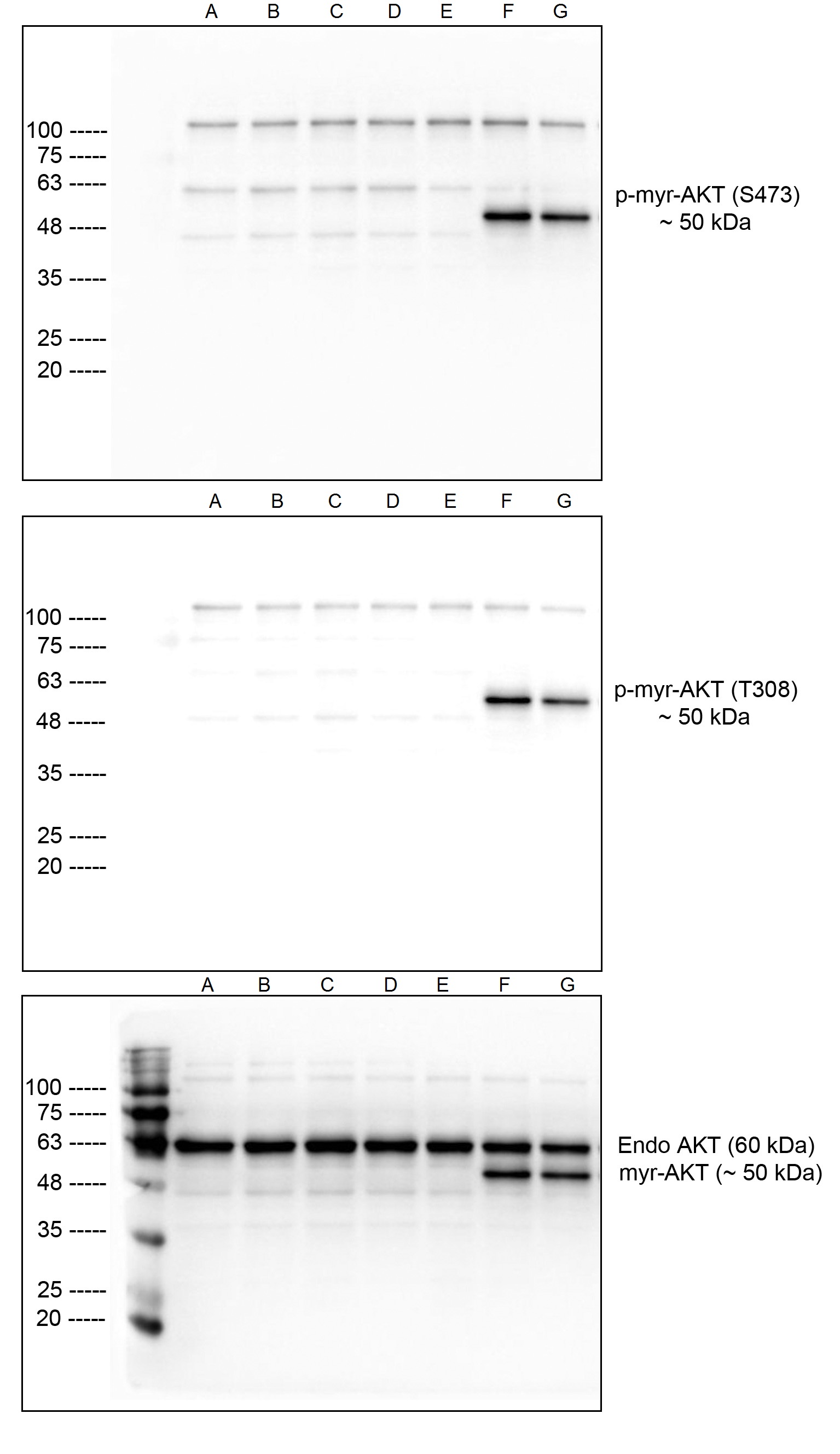


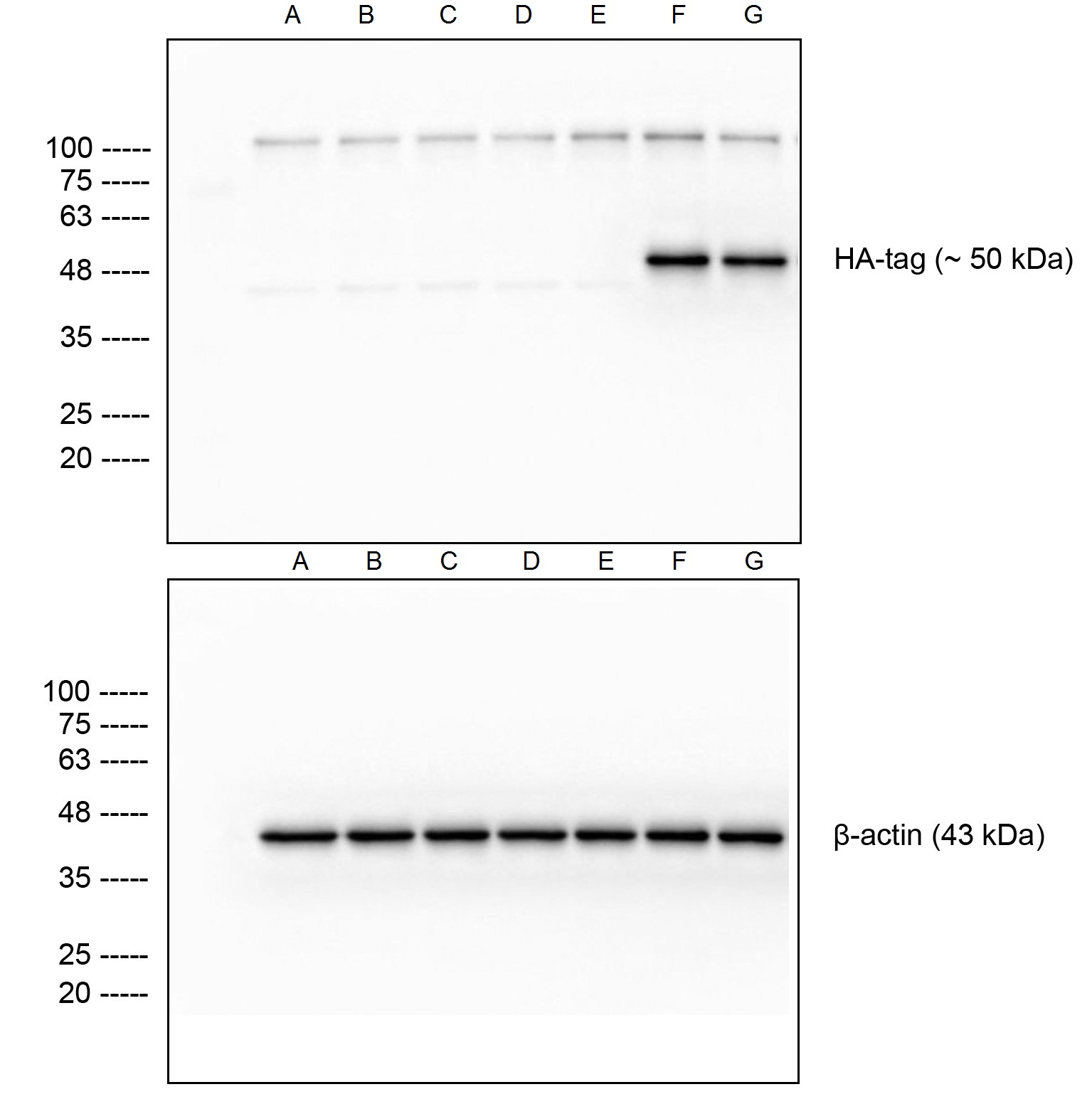


Representative Blot Images for Figure 6d

A - Negative control (transfected without plasmids)

B - Negative control (no transfection, with empty vector - pECE)

C - Negative control (no transfection, with myr-AKT)

D - Negative control (no transfection, with empty vector, with myr-AKT)

E - Empty vector (transfected with pECE)

F - Constitutively active AKT (transfected with myr-AKT)

G - Constitutively active AKT (transfected with myr-AKT, treatment with tHGA)


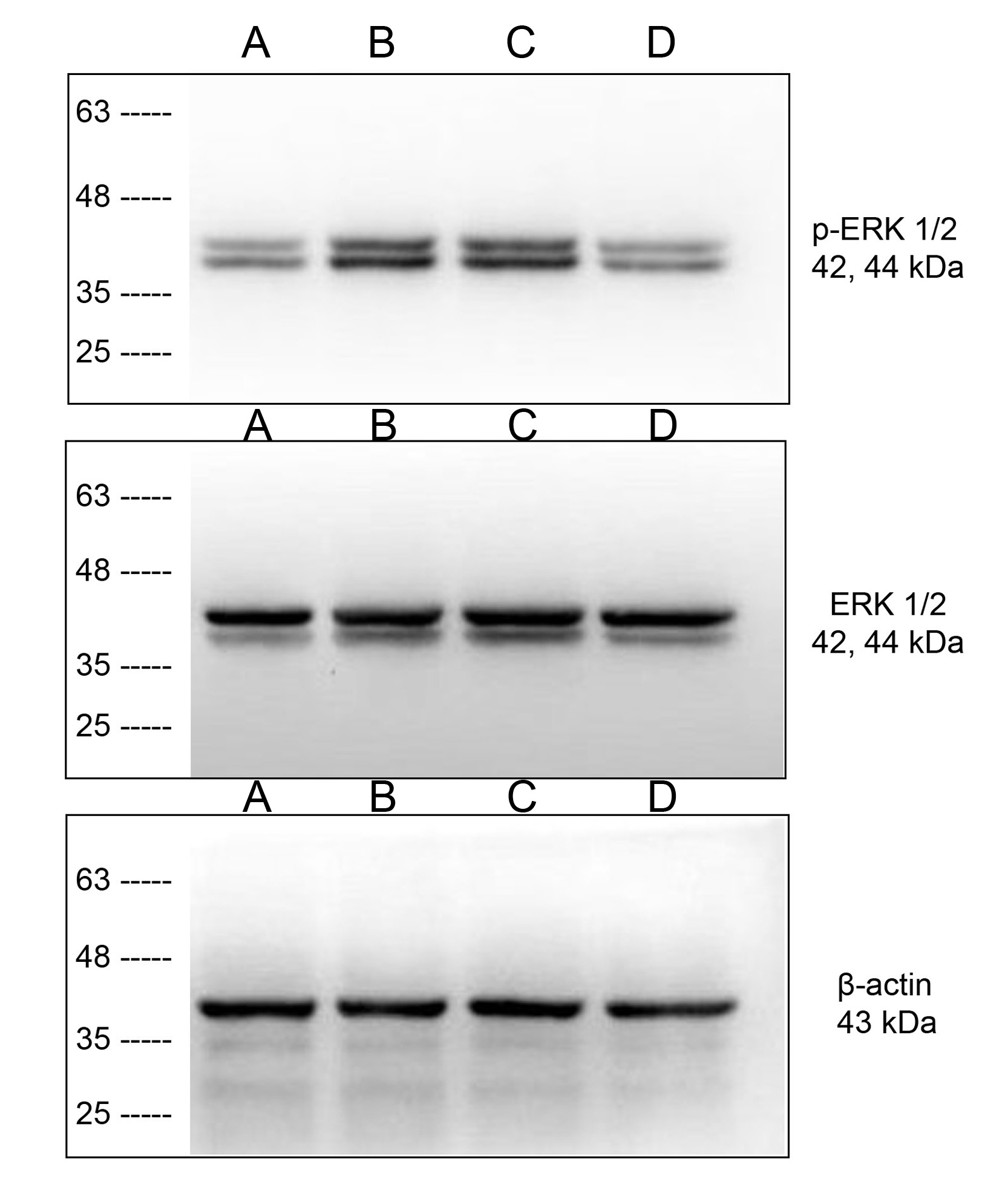


Representative Blot Images for Supplementary Figure S1a

A – Untreated hBSMCs

B – hBSMCs induced with growth factor

C – hBSMCs induced with growth factor + 20 µM tHGA

D – hBSMCs induced with growth factor + 25 µM PD98059


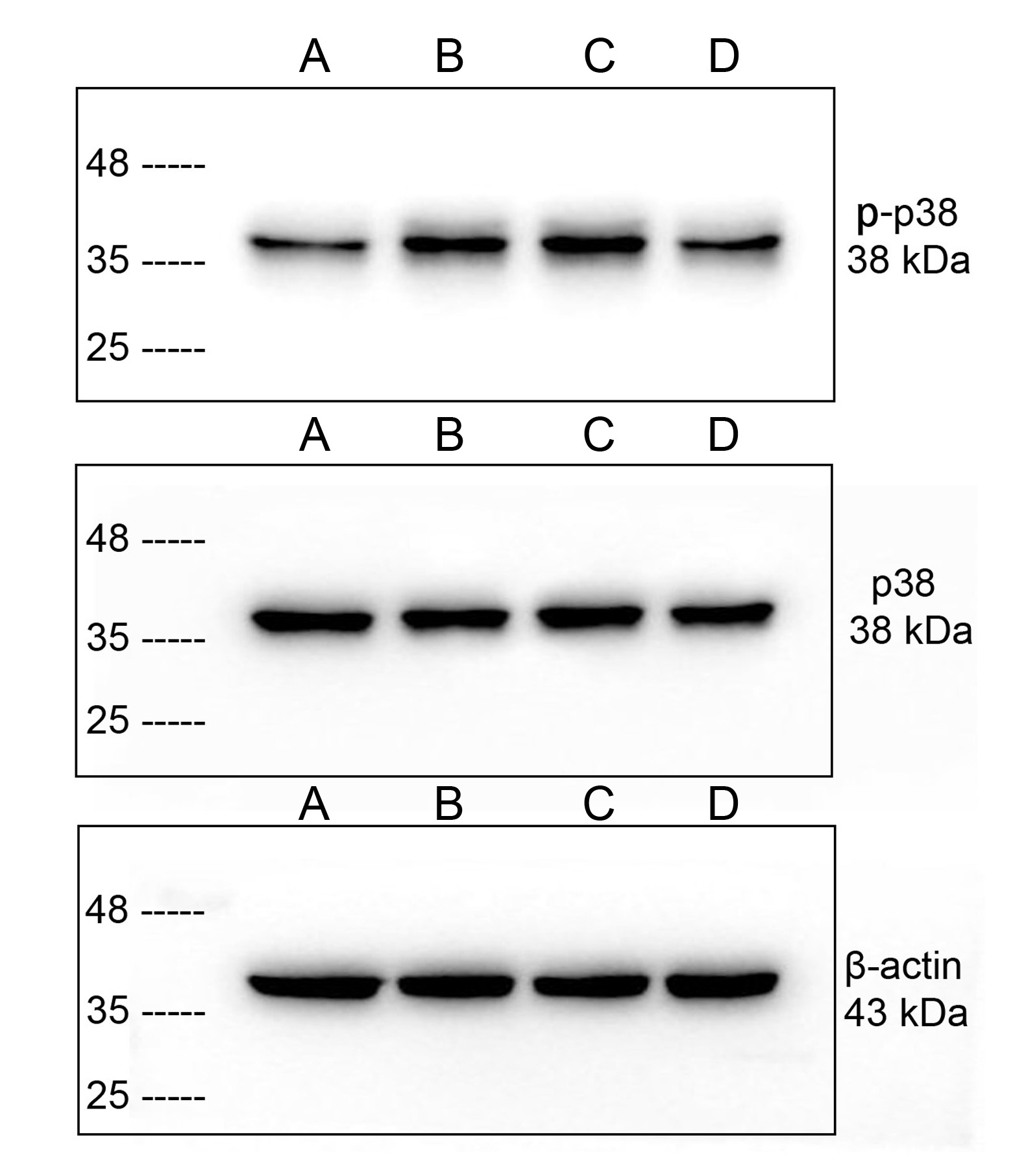


Representative Blot Images for Supplementary Figure S1b

A – Untreated hBSMCs

B – hBSMCs induced with growth factor

C – hBSMCs induced with growth factor + 20 µM tHGA

D – hBSMCs induced with growth factor + 10 µM SB202190


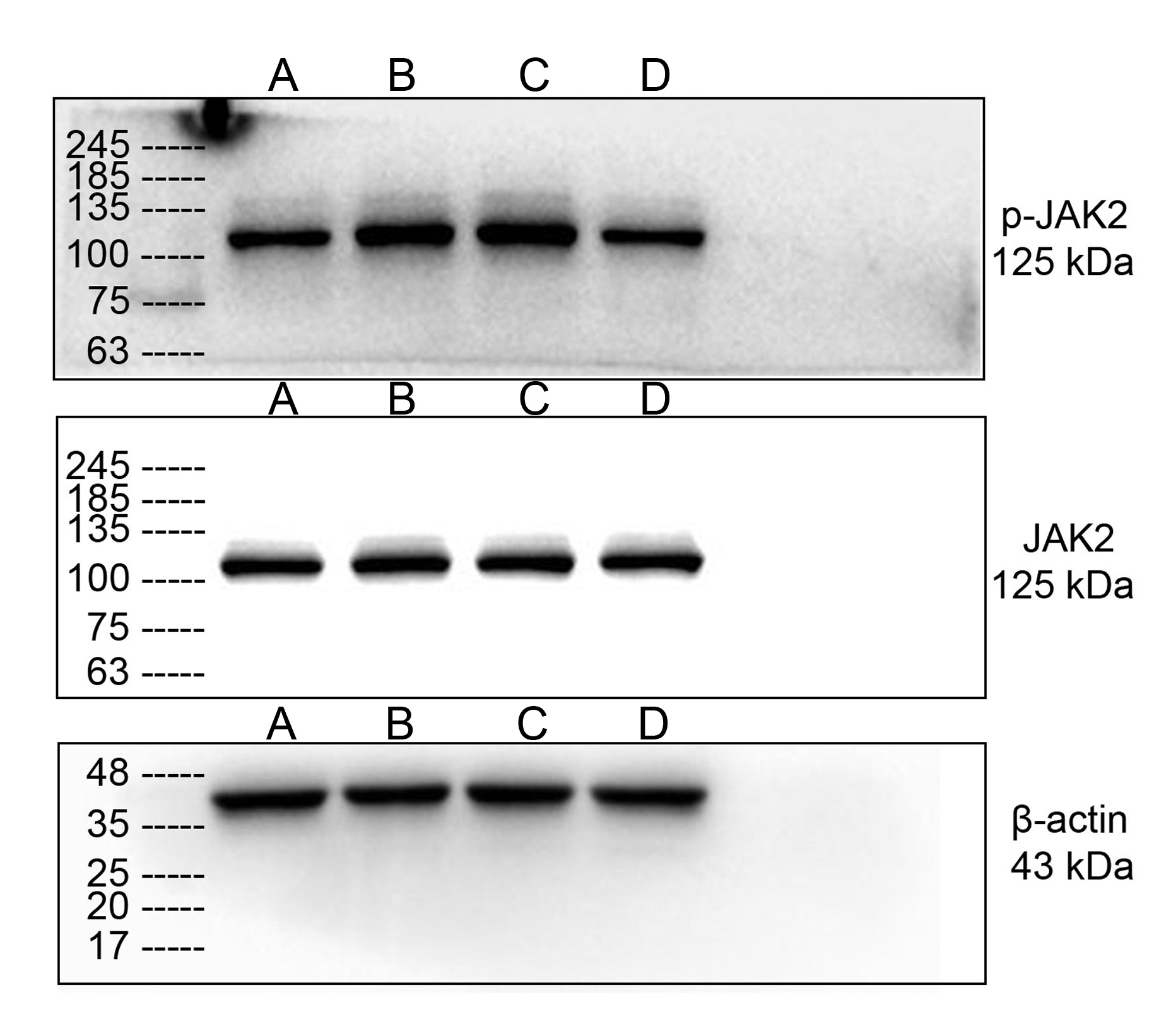


Representative Blot Images for Supplementary Figure S1c

A – Untreated hBSMCs

B – hBSMCs induced with growth factor

C – hBSMCs induced with growth factor + 20 µM tHGA

D – hBSMCs induced with growth factor + 50 µM AG490


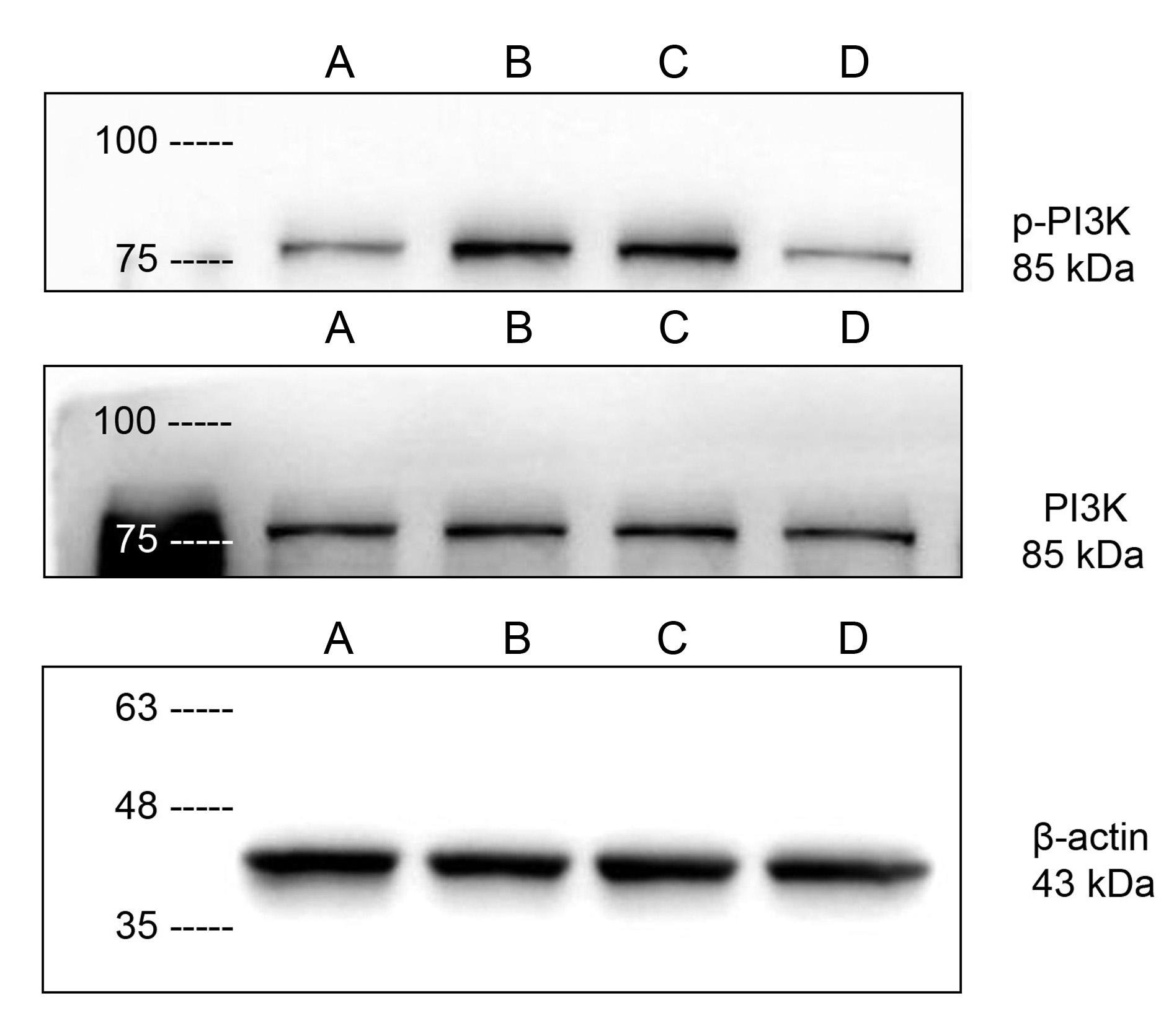


Representative Blot Images for Supplementary Figure S2a

A – Untreated hBSMCs

B – hBSMCs induced with growth factor

C – hBSMCs induced with growth factor + 20 µM tHGA

D – hBSMCs induced with growth factor + 10 µM LY294002


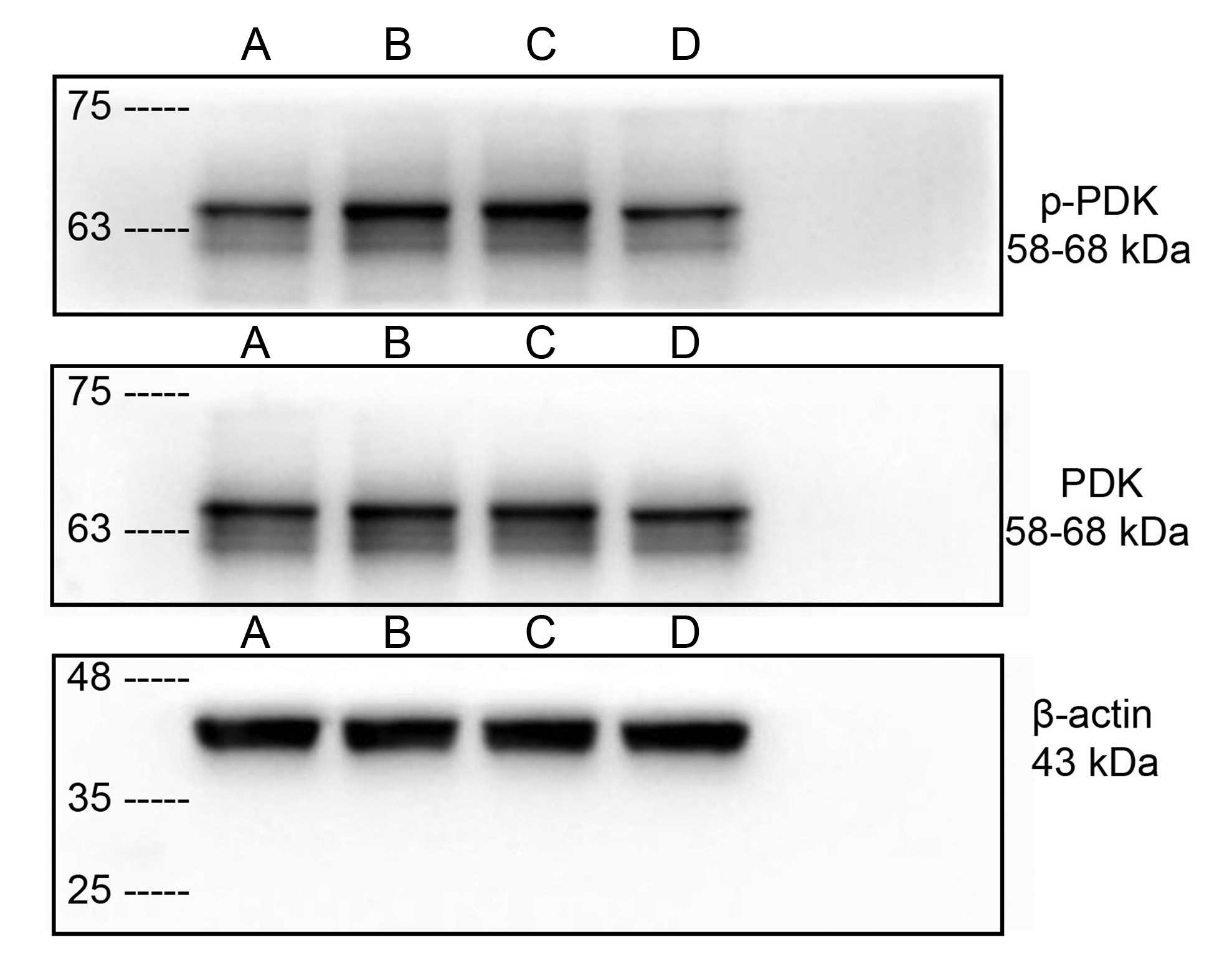


Representative Blot Images for Supplementary Figure S2b

A – Untreated hBSMCs

B – hBSMCs induced with growth factor

C – hBSMCs induced with growth factor + 20 µM tHGA

D – hBSMCs induced with growth factor + 10 µM BX795


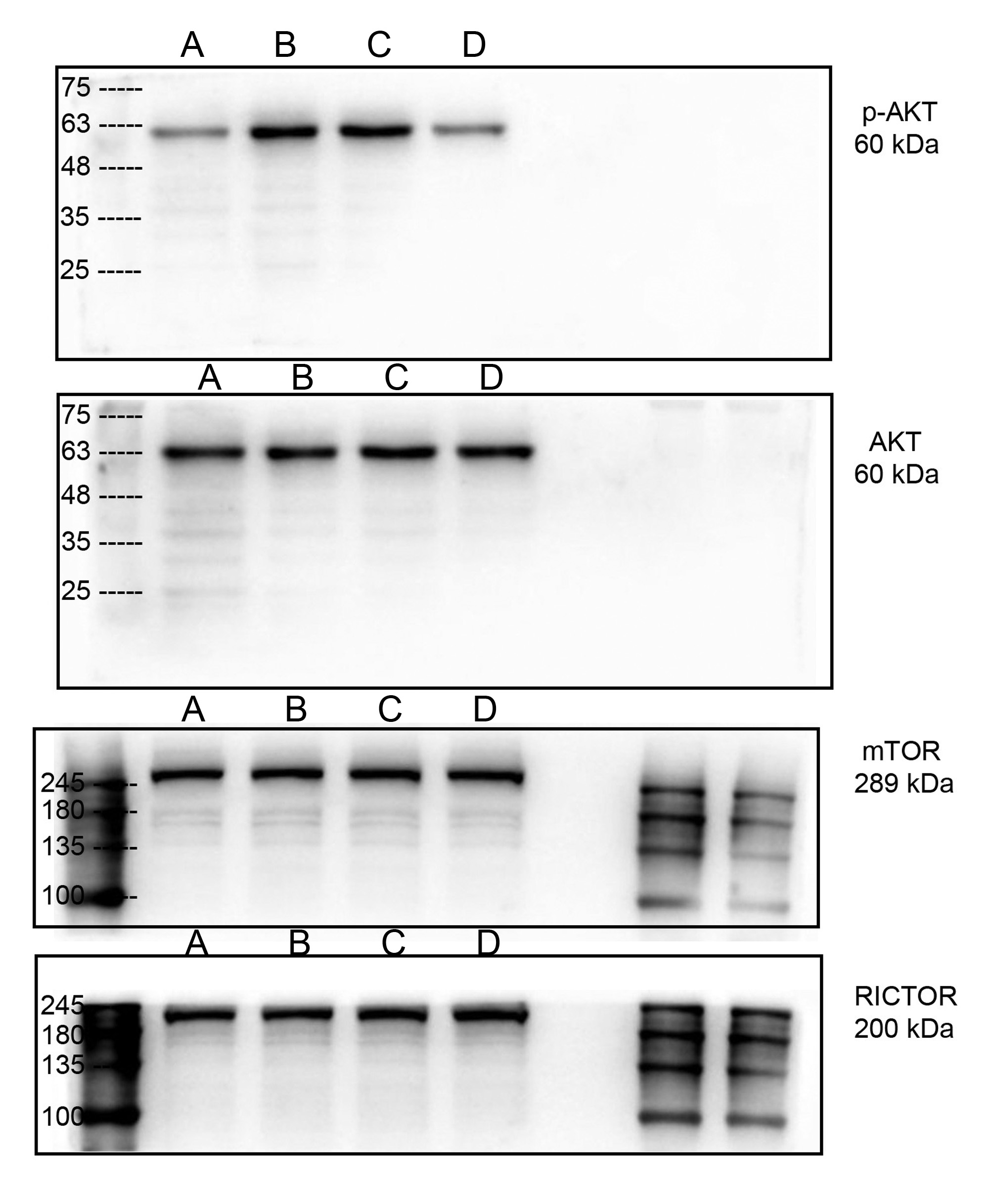


Representative Blot Images for Supplementary Figure S2c

A – Untreated hBSMCs

B – hBSMCs induced with growth factor

C – hBSMCs induced with growth factor + 20 µM tHGA

D – hBSMCs induced with growth factor + 10 µM LY294002


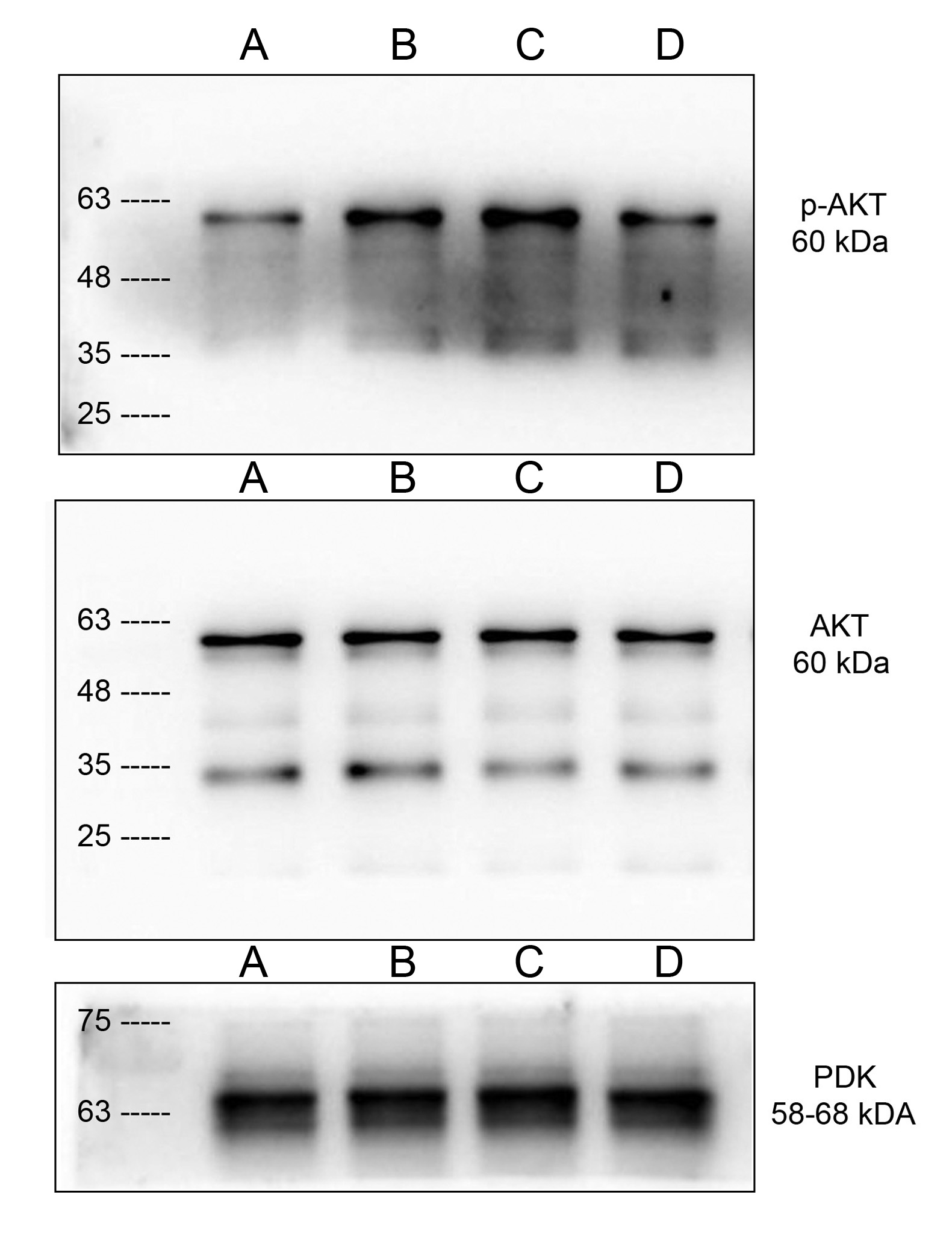


Representative Blot Images for Supplementary Figure S2d

A – Untreated hBSMCs

B – hBSMCs induced with growth factor

C – hBSMCs induced with growth factor + 20 µM tHGA

D – hBSMCs induced with growth factor + 10 µM BX795
